# Control of *Aedes albopictus* populations by silencing of the vesicular GABA transporter (*vgat*) and the vesicular monoamine transporter (*vmat*) genes using recombinant *Chlorella* shRNA

**DOI:** 10.1186/s13071-025-07053-x

**Published:** 2025-10-14

**Authors:** Xiaodong Deng, Chunmei Xue, Jingwen Gao, Xiaodan Huang, Dianlong Xu, Juncai Li, Xiaowen Fei

**Affiliations:** 1 Department of Biochemistry and Molecular Biology & Key Laboratory of Tropical Translational Medicine of Ministry of Education, College of Basic Medical Sciences, Hainan Medical University, Haikou, 571199 China; 2 Institute of Tropical Bioscience and Biotechnology, Science & Key Laboratory of Biology and Genetic Resources of Tropical Crops of Hainan Province, Chinese Academy of Tropical Agricultural, Hainan Institute for Tropical Agricultural Resources, Haikou, 571101 China; 3 College of Life Sciences and Agriculture, Jiamusi University, Jiamusi, China; 4 Hainan Provincial Key Laboratory for Functional Components Research and Utilization of Marine Bio-Resources, Haikou, China; 5 Zhanjiang Experimental Station, CATAS, Zhanjiang, 524013 China

**Keywords:** RNA interference (RNAi), *Chlorella vulgaris*, Attractive toxic sugar baits(ATSB), Non-target organisms, Biocide

## Abstract

**Background:**

*Aedes albopictus* is a primary vector for the transmission of dengue fever. RNA interference (RNAi)-based biocidal technology represents an important alternative and complement to conventional chemically synthesized insecticides.

**Methods:**

*Chlorella vulgaris* was used as a carrier organism for RNAi-mediated gene silencing of *Ae. albopictus*. Short hairpin RNA (shRNA) expression vectors targeting the *vgat* and *vmat* genes of *Ae. albopictus* were constructed and subsequently used to transform *C. vulgaris*. The shRNA-transformed *Chlorella* were then administered to *Ae. albopictus* larvae or incorporated into attractive toxic sugar baits (ATSB) for adult feeding. The effects of silencing the *vgat* and *vmat* genes on *Ae. albopictus* were then investigated.

**Results:**

Both *vgat* and *vmat* shRNA-recombinant *Chlorella* exhibited high lethality against *Ae. albopictus* larvae and adults. Conversely, shRNA-recombinant *Chlorella* ATSBs were found to have no lethal effects on non-target organisms, including *Drosophila melanogaster*, *Megalurothrips usitatus*, *Messor structor*, and *Lithobates catesbeiana* tadpoles. Together, these results confirm the specificity and safety of using shRNA-recombinant *Chlorella* as a mosquito-killing agent. The results of a semi-field trial demonstrate that recombinant *Chlorella* ATSB maintained high lethality against *Ae. albopictus* and significantly reduced the number of eggs laid by this species. Additional experiments revealed that knockdown of the *vgat* gene in *Ae. albopictus* resulted in reduced sleep duration, leg twitching, and impaired flight. Conversely, knockdown of the *vmat* gene increased sleep duration, impaired walking and flight, and induced insensitivity to light levels.

**Conclusions:**

Recombinant *Chlorella* expressing *vgat* and *vmat* shRNAs demonstrated strong lethality, high specificity, and good safety against both larval and adult *Ae. albopictus*. Oral delivery of shRNAs effectively knocked down mosquito target genes, providing a convenient approach for functional studies of mosquito genetics. Notably, this study is an important addition to the use of recombinant microalgae as a mosquito biocide.

**Graphical Abstract:**

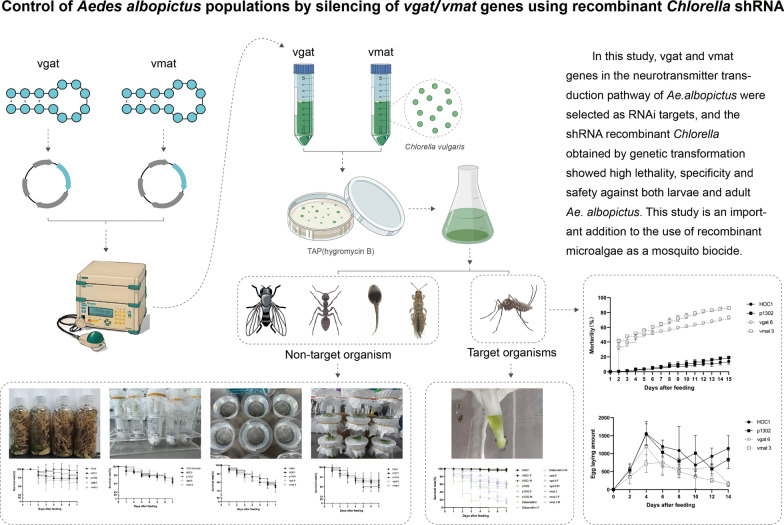

**Supplementary Information:**

The online version contains supplementary material available at 10.1186/s13071-025-07053-x.

## Background

*Aedes* mosquitoes play a central role in the transmission of mosquito-borne diseases. Several major mosquito-borne diseases associated with global epidemics, including dengue fever, Zika virus disease, yellow fever, and chikungunya, are transmitted by *Aedes* mosquitoes. The main pathogen-carrying *Aedes* mosquitoes are *Aedes aegypti* and *Ae. albopictus* [1–3]. Because an effective drug treatment for dengue fever is not currently available, governments worldwide are reliant on vector control to prevent and control dengue epidemics. To date, insecticides have mainly been used to control the *Aedes* mosquito. However, the spraying of insecticides causes irreversible pollution of the environment. Moreover, mosquitoes are becoming increasingly more tolerant of common insecticides. Hence, there is an urgent need for an environmentally friendly biocidal mosquito technology to achieve control of *Aedes* populations [4–8].

The application of biotechnology to control vector insect populations to reduce associated infectious diseases is an important direction in the management of mosquito-borne diseases. Thus far, techniques to induce Wolbachia infection or male sterility through genetic modification of mosquitoes have demonstrated some progress in this area [9–18]. However, these strategies are costly because of the large-scale cultivation and meticulous screening of male mosquitoes in specialized facilities. Microalgae, which serve as a natural aquatic food source for mosquito larvae, are abundant in ponds and freshwater streams in mosquito habitats [19–22]. Moreover, species such as *Chlorella, Dunaliella, Spirulina*, and *Chlamydomonas* can be produced industrially on a large scale. Compared with Wolbachia-based approaches or the application of genetically modified *Aedes* mosquitoes with induced male sterility, the large-scale production of microalgae is both cost-effective and manageable. When released into confined water bodies, these microalgae can quickly establish dominance and gradually reduce the local *Aedes* population. This innovative strategy offers a promising alternative for the biological control of mosquitoes, potentially reducing the spread of serious infectious diseases such as dengue fever, zika virus disease, and yellow fever [22–25].

The insect central nervous system (CNS) is the primary target of all four classes of insecticides, that is, pyrethroids, organophosphates, carbamates, and organochlorines [26–28]. Because prolonged opening of sodium channels and neuronal overstimulation can both cause insect death, the CNS provides ample opportunity to explore novel targets for insecticide design using genetic approaches. γ-Aminobutyric acid (GABA) is a neuroleptic in the nervous systems of both mammals and insects (including some parasitic helminths), and this inhibitory neurotransmitter has an important effect on the level of neuronal excitation [29–32]. The vesicular GABA transporter (VGAT) is situated in the vesicular membrane, where it plays an important role in maintaining the stability of intracellular and extracellular GABA concentrations, GABA release, and recycling. In the GABAergic transmission system, GABA is translocated into vesicles via the VGAT and transported to the presynaptic membrane. When GABAergic neurons receive a stimulus signal, GABA at the presynaptic membrane is released into the synaptic gap, and it subsequently binds to GABA receptors on the postsynaptic membrane to mediate neural signaling [33–35].

The behavior of insects is extensively regulated by monoamine neurotransmitters [36–42], including (in *Drosophila melanogaster* and most other insects) 5-HT, dopamine (DA), histamine, octopamine (OA), and tyramine (TA) [43–51]. These monoamine neurotransmitters are transported into vesicles via the vesicular monoamine transporter protein (VMAT) in the vesicular membrane, which maintains a stable concentration of monoamine neurotransmitters in neuronal cells. In the dopaminergic system (as an example), dopamine is transported into vesicles via VMAT and, upon stimulation, they are transported to the presynaptic membrane where DA is released into the synaptic cleft; there, it subsequently binds to dopamine receptors in the postsynaptic membrane to mediate neural signaling. Following signaling, DA from the synaptic cleft is rapidly taken up by the dopamine transporter protein (DAT), which works with VMAT to replenish vesicular dopamine stores. Hence, key proteins of the dopaminergic system, including DAT and dopamine receptors, are important targets for the action of chemical insecticides.

Vesicular transporter proteins are members of the solute carrier (SLC) family, containing multiple transmembrane structural domains, and these proteins are responsible for the movement of relatively small (~ 75–200 Da) hydrophilic molecules across lipid membranes. As 'active' transporter proteins, SLCs use energy to drive the movement of neurotransmitters against a concentration gradient [52–54]. In insects, VMAT is known to be a member of the SLC18 subfamily, whereas VGAT belongs to the SCL32 subfamily [55–58]. Given the importance of VGAT and VMAT in insect neural signaling, VGAT and VMAT are crucial targets in the development of novel insecticides [59, 60].

Our previous research has used microalgae expressing dsRNA-interfering fragments targeting the *Ae. aegypti HR3*, *3HKT*, and *CHSA* genes to effectively control the *Ae. aegypti* mosquito population [22–25]. However, studies have shown that sustained selective pressure for silencing specific gene targets can lead to mosquito tolerance (drug resistance) against the target RNAi-interfering algal strains [61–63]. Therefore, it is essential to identify novel RNAi targets within distinct metabolic pathways that can serve as alternatives when existing targets become ineffective. This strategy could also lead to the development of more potent biological insecticides with enhanced lethal efficacy. In the present study, we selected the *Ae. albopictus vgat* and *vmat* genes as RNAi targets and constructed shRNA expression vectors to transform *Chlorella*. The shRNA-recombinant *Chlorella* were then administered orally to *Ae. albopictus* larvae. To treat adult mosquitoes, spiked sugar baits were also prepared. The effects of *vgat* and *vmat* gene silencing on *Ae. albopictus* larvae and adults were then systematically evaluated. In addition, recombinant *Chlorella* with potent lethality against *Ae. albopictus* were screened to provide experimental evidence for the development of environmentally friendly insecticides and innovative strategies for the prevention and control of mosquito-borne diseases.

## Methods

### Bioinformatic analysis of protein orthologs

Comprehensive bioinformatic analyses were performed to identify orthologs of VGAT and VMAT across a range of species. The *Ae. albopictus* VGAT and VMAT protein sequences were used as queries in BLASTP searches against the VectorBase (https://vectorbase.org/vectorbase/app) and NCBI (National Center for Biotechnology Information) databases (using default parameters). Conserved DNA motifs were predicted using the online MEME tool. Protein domain architecture was analyzed using InterProScan. Phylogenetic reconstruction was performed in MEGA11 using the neighbor-joining (NJ) algorithm with Poisson-corrected distances and pairwise deletion of gaps. Finally, schematic representations of DNA motif and protein domain organization were generated by integrating the MEME and InterProScan results with manual curation of sequence features.

### Cultivation of microalgae and mosquito maintenance

The *Chlorella vulgaris* HOC1 strain was isolated from local water sources in Hainan (China) and cultured in TAP medium. Liquid cultures were maintained at 25 °C with shaking at 180 rpm under continuous light (150 µmol/m^2^·s) [23].

Wild-type *Ae. albopictus* were captured in Haikou (China) and reared in the laboratory for six generations. All specimens were maintained in the insectary of the Chinese Academy of Tropical Agricultural Sciences as described in previous studies [24, 25]. Adults were fed 10% sucrose-soaked sponge blocks. After mating, females were provided with blood meals using splint-fixed rats (for 5 h each day) to stimulate egg-laying. A moistened filter paper placed in a petri dish served as an oviposition site. The egg-laden papers were subsequently dried and stored for later use.

### Design of *vgat *and* vmat* shRNAs and construction of expression vectors

Starting with the *Ae. albopictus vgat* and *vmat* gene sequences (AALF004203, AALFPA057308) available on VectorBase, high-scoring shRNA target sequences were screened using the online GPP portal (https://portals.broadinstitute.org/ gpp/public/). Target sequences with high specificity were selected after excluding homologs using NCBI BLAST. After selection, cleavage sites for BglII and BstEII were added to the ends (via a manual process). shRNA secondary structure predictions for the selected sequences were performed using the Mfold web server (http://www.unafold.org/) (Additional file 1: Fig. S1). After subsequent synthesis (BGI Genomics Co., Ltd., Shenzhen, China), the selected shRNAs were cloned into the pCAMBIA1302 expression vector to yield pCAMvgat-shRNA and pCAMvmat-shRNA. The final constructs were then verified by sequencing.

### Chlorella transfection

*Chlorella* cultures were collected via centrifugation and resuspended in electroporation buffer. For transfection, 2 µg of plasmid (pCAMvgat-shRNA or pCAMvmat-shRNA) was mixed with the cells, incubated on ice for 15 min, and then electroporated in a BTX ECM 630 electroporator (1600 V/cm, 1 ms pulse). The suspension was subsequently transferred to TAP medium with 60 mM sorbitol and incubated overnight. The transfected cells were then collected by centrifugation (4000 × g, 3 min) and plated on TAP agar containing 10 µg/ml hygromycin. The colonies appeared within 5–10 days [64, 65].

### Mosquito feeding experiments

Following transfection, PCR was used to confirm shRNA integration in transgenic *Chlorella*. Positive clones were fed to mosquitoes in insectary experiments following previously described methods [25]. Initial experiments tested feeding concentrations of 0.25, 1, 5, 15, and 25 mg/ml of *Chlorella*, with 10 L1 larvae per group (in triplicate). The optimal feeding concentration was determined based on larval mortality.

For formal trials, larvae were split into treatment and control groups (30 L1 larvae in 20 mL water with 300 mg *Chlorella* at 15 mg/ml). Treatment groups were fed transgenic *Chlorella* previously transfected with pCAMvgat-shRNA or pCAMvmat-shRNA. Controls received wild-type or empty-vector *C. vulgaris*. Larval mortality, pupation, and adult emergence were recorded across three replicates. The most lethal recombinant *Chlorella* strains were selected for further study.

### RT-qPCR

Ten to 20 L3 larvae or 15 adult mosquitoes (after 3 days of ATSB feeding) were pooled per group. Total RNA was extracted using TRIzol (Takara, Japan) and reverse transcribed into cDNA using oligo-dT primers (Shenggong, China). Real-time PCR was performed with SYBR Green on a BioRad iCycler iQ system (BioRad, USA). The *Ae. albopictus* RPS17 gene was used as an internal reference. Primers for *vgat*, *vmat*, and clathrin-mediated endocytosis (CME) pathway genes are listed in Additional file 1: Tables S1 and S2. Relative expression levels were calculated using the 2^−ΔΔCT^ method [66], with triplicate measurements and normalization to the internal control [67].

### Attraction test of natural attractants on adult mosquitoes

To evaluate sugar-based attractants, juices from six fruits (mango, citrus, mulberry, dragon fruit, tomato, and poplar peach) were tested against 10% sucrose as a control. Fruit juices were obtained by homogenizing and filtering fruit purchased in Haikou, China. Each 10-ml juice sample was mixed with *C. vulgaris* (15 mg/ml) and applied to bait devices (cotton + Eppendorf tube). These baits were then suspended in the corners of cages containing 50 starved adult mosquitoes. The number of times *Ae. albopictus* adult mosquitoes landed and then fed on the sugar bait was subsequently counted over a 30-min period (starting when the first *Ae. albopictus* sucked the sugar bait).

### Attractive toxic sugar bait (ATSB) test

The ATSB feeding protocol was modified from that of Hapairai et al. [68]. ATSBs were prepared by mixing 30 mg of transgenic *Chlorella* with 2 ml 10% sucrose. Controls included wild-type *Chlorella*, empty-vector transformed *Chlorella*, and 0.1 mg/l deltamethrin in sucrose (positive control). Baits were delivered in cotton-filled Eppendorf tubes (bottom open) suspended in mosquito cages (30 × 30 × 30 cm). Fifty mosquitoes (50% male, 50% female), starved for 24 h, were placed in each cage. All baits were refreshed daily. The sex-specific mortality was recorded daily, and target gene expression was evaluated after 3 days of feeding. All tests were performed in triplicate.

### ATSB test for non-target organisms

*Drosophila melanogaster*, *Messor structor*, *Megalurothrips usitatus*, and *Lithobates catesbeiana* (tadpoles; chosen to test the effects of shRNA recombinant *Chlorella* on aquatic animals) were selected to determine whether toxic sugar baits have an effect on non-target organisms.

(i) *Drosophila melanogaster* survival test:

The test was conducted at 22 °C under a 12-h light/12-h dark photoperiod. *Drosophila melanogaster* were fed a basal medium. The control groups received either *Chlorella vulgaris* HOC1 or *Chlorella* transformed with pCAMBIA1302 (15 mg/ml), both mixed with 10% sucrose in the basal medium. The treatment groups received shRNA recombinant *Chlorella* (15 mg/ml) mixed with 10% sucrose in the basal medium. Fifty *D. melanogaster* were used per group. Bait consumption was confirmed by observing red food dye in the intestines of the flies. Flies were considered dead if they showed prolonged immobility and were lying on their backs or sides in the rearing bottle. Mortality was recorded over a 7-day period, and the experiment was repeated three times.

(ii) *Messor structor* survival test:

Control ants were fed sponge blocks (1.5 × 1.5 × 1.5 cm) soaked with *C. vulgaris* HOC1 (15 mg/ml) mixed with 10% sucrose and 5% red food dye. The treatment group received sponge blocks soaked with shRNA recombinant *Chlorella* (15 mg/ml) mixed with 10% sucrose. Ants were housed in 50-ml tubes sealed with gauze, and the sponge blocks were replaced every 24 h. Each group contained 50 ants. Ants were considered dead if they ceased movement. Mortality was recorded over a 7-day period, and the experiment was repeated three times.

(iii) *Megalurothrips usitatus* survival test:

*Megalurothrips usitatus* were fed Dutch beans (purchased from RT-Mart in Haikou). All beans were washed and rinsed with sterile water prior to feeding. In the control group, flies were fed either untreated beans or beans soaked for 12 h in a mixture of *C. vulgaris* HOC1 (15 mg/ml) and 10% sucrose to enhance algal adhesion. The treatment group received beans soaked for 12 h in a mixture of shRNA recombinant *Chlorella* (15 mg/ml) and 10% sucrose. Treated beans were replaced daily. Each group included 20 flies. Inactivity upon gentle brush contact was used as the criterion for death. Mortality was recorded over a 7-day period, and the experiment was repeated three times.

(iv) *Lithobates catesbeiana* tadpole survival test:

To prepare tadpole feed, shRNA recombinant *Chlorella* was inoculated into TAP medium, and this small-scale culture was scaled up to a 10 l culture. Following incubation, the culture was centrifuged at 8000 rpm for 10 min. After removing the supernatant, the algal sludge was collected and mixed with commercial tadpole feed at a concentration of 15 mg/g. Control groups were fed either plain feed or *C. vulgaris* HOC1 mix, while treatment groups received the shRNA recombinant *Chlorella* mix. Tadpoles were housed in disposable 1-l bowls containing 500 ml water. Each group consisted of 40 tadpoles (approximately 1 cm in length), and these were provided 30 mg of feed per day. Water quality was monitored and changed as needed to maintain clarity. Tadpoles were considered dead if they stopped moving in the water. Mortality was recorded over a 7-day period, and the experiment was repeated three times.

### Semi-field trial

To simulate the lethal effect of ATSB on mosquitoes in a natural setting, a semi-field ATSB experiment was conducted (during the summer of 2023) in a mosquito culture room located on the roof of the experimental building at the Institute of Tropical Biotechnology, Chinese Academy of Tropical Agricultural Sciences (CATAS). This experiment was performed using a modified version of the method described by Stewart et al. [69]. During the test period, the ambient temperature ranged from 25 °C to 35 °C, and the average humidity was 70 ± 15%.

Two sponge blocks (2 × 2 × 2 cm), one impregnated with shRNA recombinant *Chlorella* and the other with 10% sucrose solution, were simultaneously placed in a mosquito rearing cage (30 × 30 × 30 cm). *Chlorella vulgaris* HOC1 and empty-vector *C. vulgaris* bait were used for the control groups, while shRNA recombinant *Chlorella* bait was used for the treatment group. Each cage contained 300 adult mosquitoes (50% male, 50% female) that had been starved for 24 h prior to feeding. The mosquitoes were allowed to feed for 15 days. The ATSB solution was replaced every 24 h. The numbers of dead mosquitoes were counted every 24 h, and the numbers of mosquitoes laying eggs were counted every 48 h. The experiment was repeated three times.

### Mosquito behavioral test

Mosquito sleep behavior was studied using the method of Ajayi et al., with slight modifications [70, 71]. The experiment was conducted in mosquito-rearing cages (30 × 30 × 30 cm), with each cage containing six 2–3-day-old adult mosquitoes housed in transparent test tubes covered at the top with fabric netting. Prior to starting the experiment, the mosquitoes were allowed to feed freely on 10% sucrose solution, but they were not provided any blood meals. On the day of the experiment, individual containers were placed within the field of view of an infrared camera connected to a computer. Once the experimenter had left the room, the mosquitoes were left undisturbed for the duration of the observation. The experimenter then remotely operated the camera to monitor activity. Using an established definition of mosquito sleep (a period of 120 min of inactivity with hind legs lowered), cumulative sleep duration was recorded under a 12-h dark/12-h light photoperiod (1700 lx) over a period of 4 days. Additionally, the walking and flight behaviors of the mosquitoes were observed and documented. This test was also repeated under conditions of high light intensity (5800 lx) to evaluate differences in light sensitivity. All experiments were replicated three times. The treatment group consisted of adult mosquitoes fed with *vgat* and *vmat* shRNA recombinant *Chlorella* baits, while the control group was fed *C. vulgaris* HOC1 baits.

### Statistical analyses

All data were analyzed using the Statistical Package for the Social Sciences (SPSS) and presented as mean ± SD (standard deviation). Duncan’s multiple range and *t*-test tests were used to examine significant differences between means. Differences with a *p*-value < 0.05 were considered statistically significant. Asterisks indicate the statistical significance in all cases: *P < 0.05; **P < 0.01; ***P < 0.001; ****P < 0.0001. Error bars indicate the standard deviation.

## Results

### VGAT and VMAT proteins

**Fig. 1:**
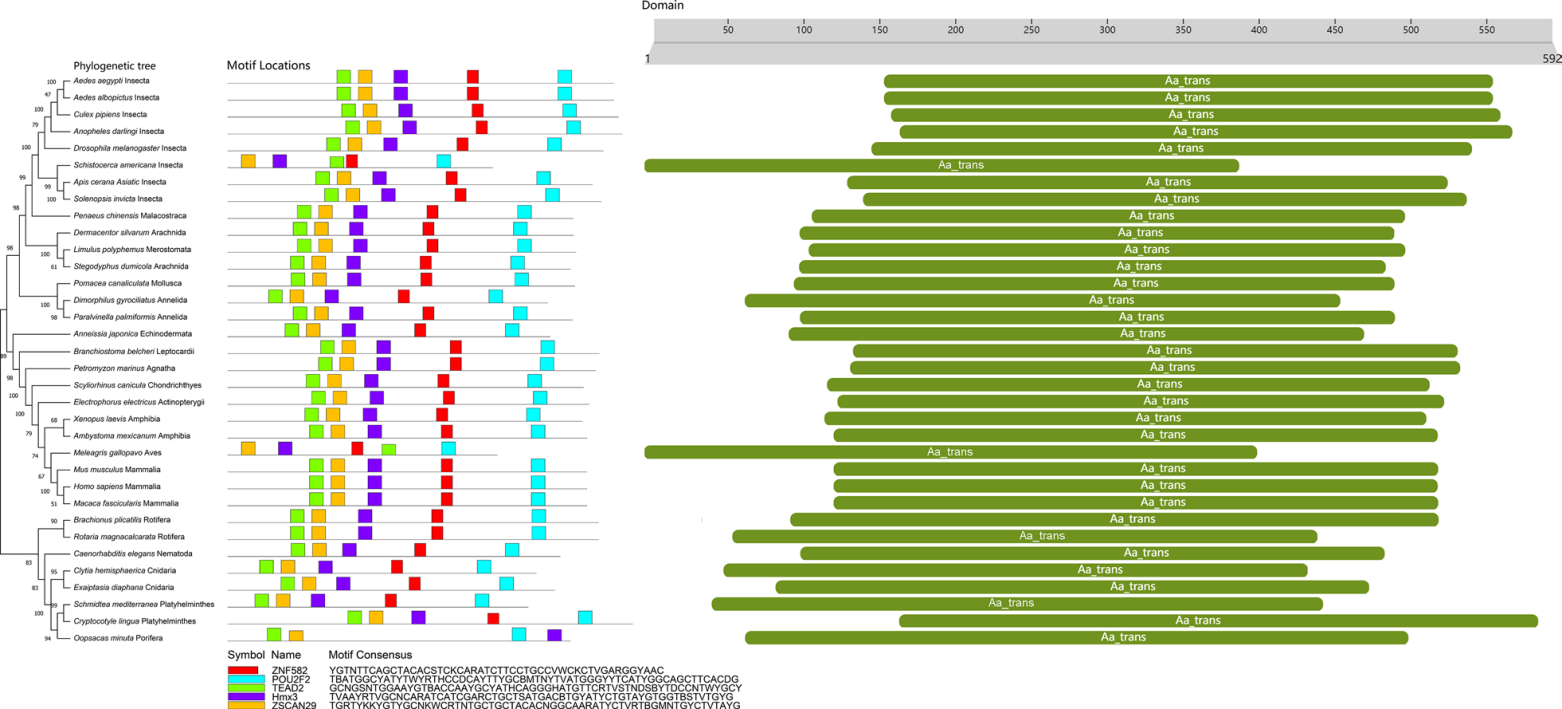
Phylogenetic relationship of VGAT and analysis of their conserved domains. **a** Phylogenetic relationship of VGAT from chordates, invertebrates (arthropods, cnidarians, porifera, annelids, platyhelminths), echinoderms, molluscs, nematodes, and rotifers. A neighbor-joining (NJ) tree was constructed using MEGA11, based on the alignment of VGAT protein sequences. Bootstrap analysis was performed with 1000 replicates. The numbers are bootstrap values based on 1000 iterations. **b** An online MEME tool (https://meme-suite.org/meme/tools/meme) was used to predict the conserved motifs of vgat genes. **c** The domains of each VGAT protein were predicted using the online InterProScan tool (http://www.ebi.ac.uk/InterProScan). Aa-trans represents the VGAT component and the amino acid transmembrane functional regions

**Fig. 2:**
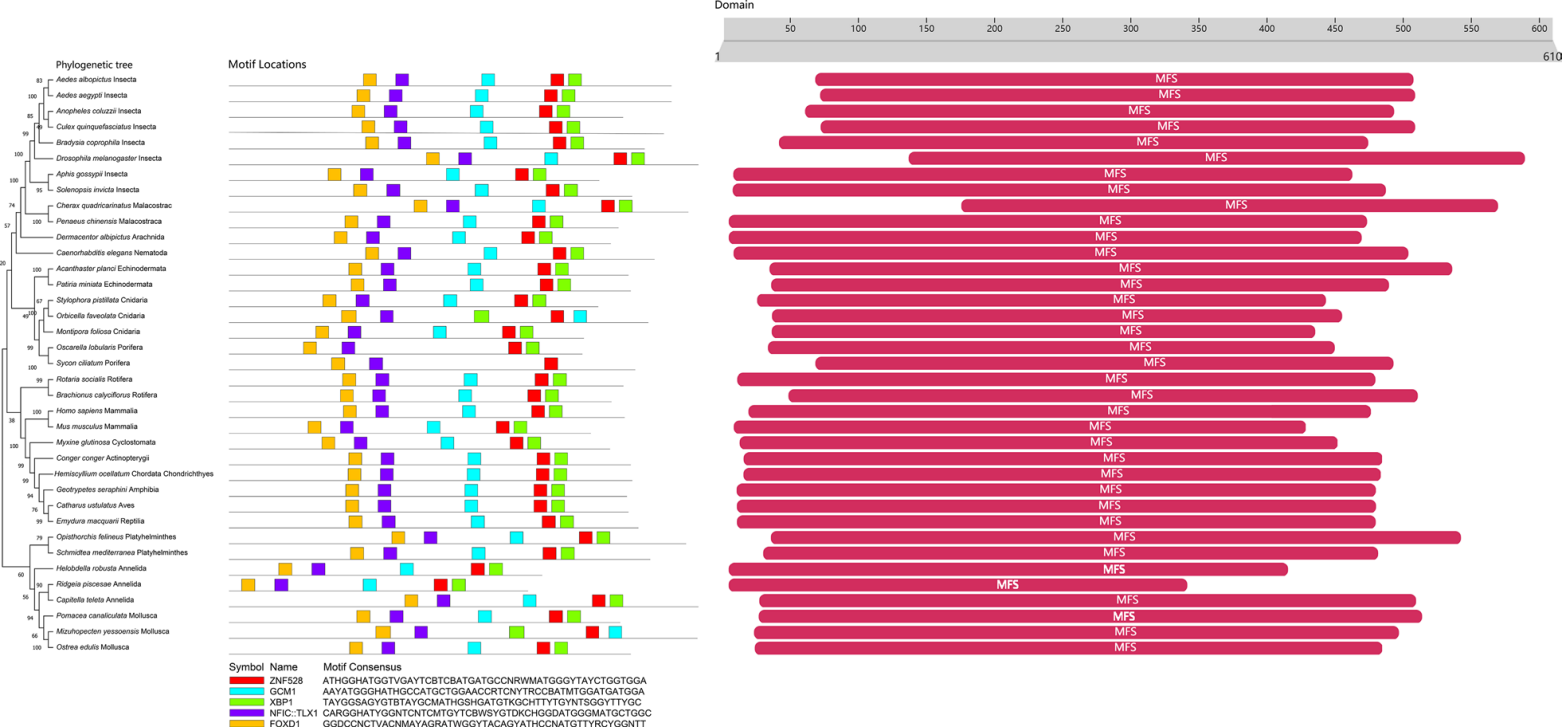
The phylogenetic relationship of VMAT and the analysis of their conserved domains. **a** Phylogenetic relationship of VMAT from chordates, invertebrates (arthropods, cnidarians, porifera, annelids, platyhelminths), echinoderms, molluscs, nematodes and rotifers. A neighbor-joining (NJ) tree was constructed using MEGA11, based on the alignment of VMAT protein sequences. Bootstrap analysis was performed with 1000 replicates. Numbers are bootstrap values based on 1000 iterations. **b** An online MEME (https://meme-suite.org/meme/tools/meme) was used to predict the conserved motifs of vmat genes. **c** The domains of each VMAT protein were predicted using the InterProScan online tool (http://www.ebi.ac.uk/InterProScan). MFS represents VMAT component, MFS functional domains

Using *Ae. albopictus* VGAT and VMAT as source sequences, a BlastP search was performed to retrieve similar amino acid sequences from a wide range of species. Several representative VGAT and VMAT orthologs from different species were subsequently selected for further analyses (Additional file 1: Tables S3 and S4), including nucleic acid and protein structural domain characterization and phylogenetic analyses (Figs. 1 and 2). An analysis of the VGAT nucleic acid sequence using the MEME website (https://meme-suite.org/meme/index.html) revealed that its coding frame contains five conserved regions, namely ZNF528 (C2H2 zinc finger factors), POU2F2 (POU domain factors), TEAD2 (tea domain transcription factors), Hmx3, and ZSCAN29 (C2H2 zinc finger factors). A similar analysis of the VMAT nucleic acid sequence revealed that its coding frame contains five conserved regions, namely ZNF528, FOXD1, GCM1, NFIC::TLX1 (SMAD/NF-1 DNA-binding domain factors), and XBP1 (basic leucine zipper (bZIP) factors). It is a reasonable postulate that one or more of these conserved regions is involved in small molecule binding. Analysis of the VGAT/VMAT protein sequences using the Interproscan online server (http://www.ebi.ac.uk/InterProScan) revealed that all VGAT proteins contain amino acid transmembrane functional regions (Aa-trans), whereas all VMAT proteins contain MFS functional domains.

VGAT and VMAT orthologs have been found in almost all animals, including the more primitive porifera (*Oopsacas minuta*, *Oscarella lobularis*, *Sycon ciliatum*) and cnidarians (*Clytia hemisphaerica*, *Exaiptasia diaphana*, *Stylophora pistillata*, *Montipora foliosa*, *and Orbicella faveolata*). To determine the evolutionary relationships between *Ae. albopictus* VGAT/VMAT sequences and those of other organisms, phylogenetic analyses of VGAT or VMAT sequences were performed. An unrooted phylogenetic tree was constructed using full-length VGAT/VMAT protein sequences from chordates, invertebrates, arthropods, cnidarians, porifera, annelids, platyhelminthes, echinoderms, molluscs, nematodes, and rotifers. As shown in Fig. 1, *Ae. albopictus* VGAT is genetically close to *Ae. aegypti*, *Culex quinquefasciatus*, *Anopheles darlingi*, and *D. melanogaster* VGAT sequences, as well as those of other arthropod species. It also forms a large branch with the mollusc *Pomacea canaliculata* and the annelids *Dimorphilus gyrociliatus* and *Paralvinella palmiformis*. *Aedes albopictus* VMAT is genetically related to *Ae. aegypti*, *Culex quinquefasciatus*, *Anopheles coluzzii*, *D. melanogaster*, *Aphis gossypii*, and *Bradysia coprophila* VMAT sequences. Additionally, *Solenopsis invicta*, *Cherax quadricarinatus*, and *Penaeus chinensis* VMAT sequences, and those of other arthropod species, are also related. Moreover, *Ae. albopictus* VMAT also forms a large branch with the nematode *Caenorhabditis elegans* (Fig. 2).

### *vgat/vmat* shRNA recombinant Chlorella are lethal to *Ae. albopictus*

**Fig. 3:**
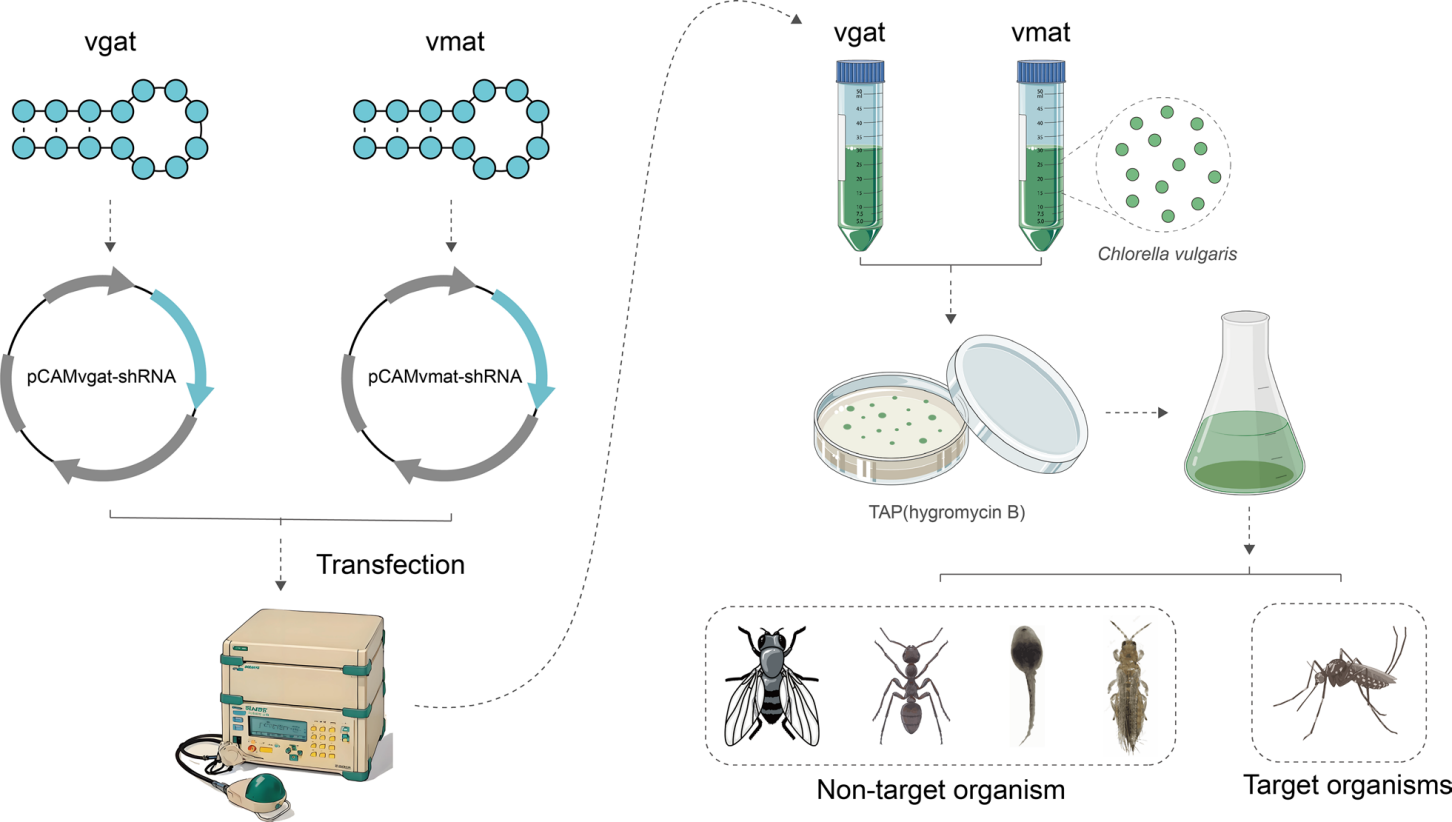
Schematic diagram of shRNA design, vector construction, and *Chlorella* transfection

The *vgat* and *vmat* genes in the neurotransmitter transmission pathway of *Ae. albopictus* were utilized as RNAi target genes (AALF004203, AALFPA057308). shRNA sequences (58 bp) for *vgat* and *vmat* were designed using the GPP website (Additional file 1: Fig. S1). Following their synthesis (by Huada Genome Technology Co.), the dsDNA segments were individually cloned into the pCAMBIA1302 expression vector. The final shRNA expression vectors obtained were termed pCAMvgat-shRNA and pCAMvmat-shRNA. Sequencing results demonstrated 100% homology with the designed shRNA sequences. The two shRNA vectors were then transformed into *C. vulgaris* HOC1, and several positive clones were identified by PCR (Additional file 1: Fig. S2) and used for subsequent experiments (Fig. 3).

**Fig. 4:**
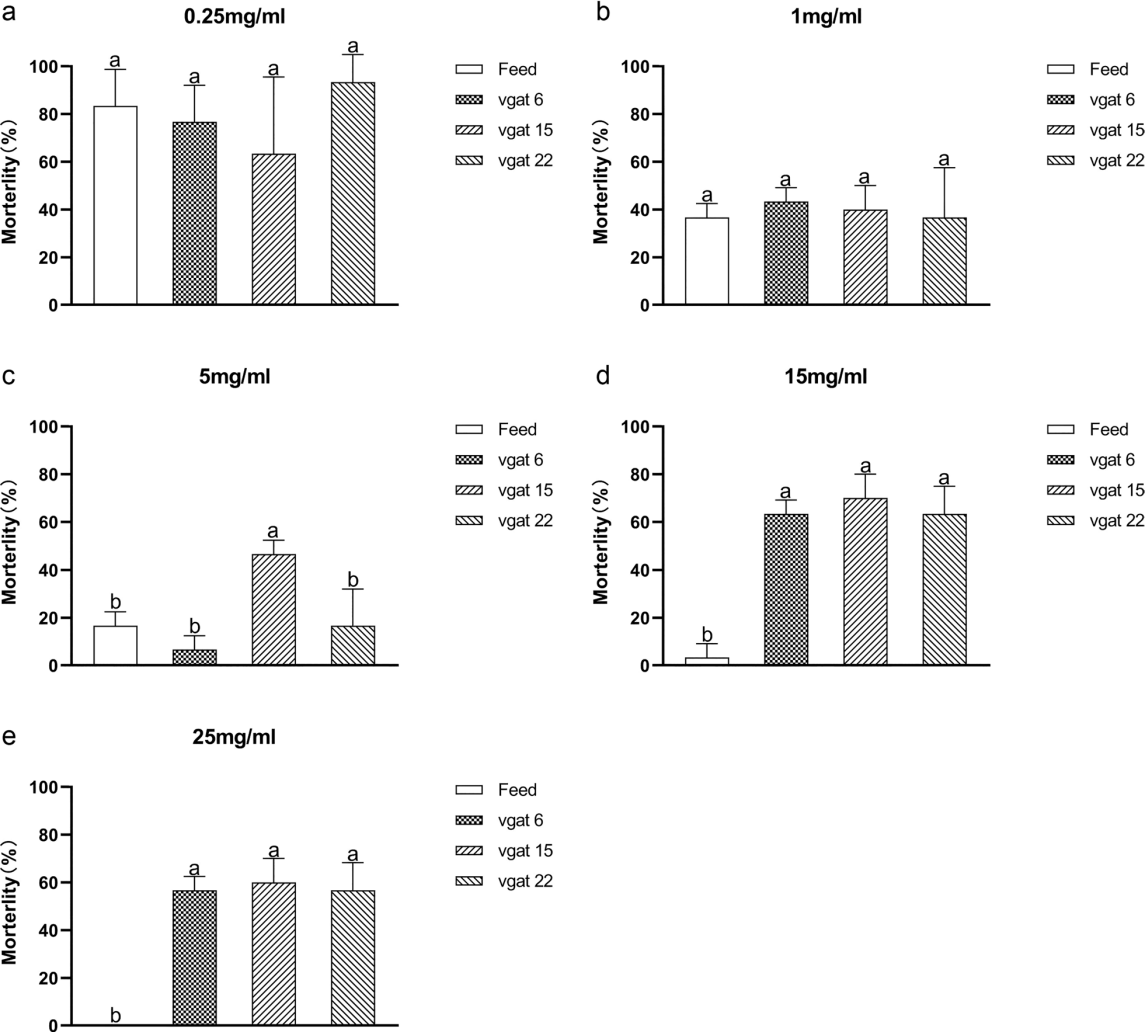
Mortality of mosquito larvae fed on vgat shRNA recombinant *Chlorella* after 15 days. **a** Larval mortality when fed 0.25 mg/ml of *vgat* shRNA recombinant *Chlorella*; **b** larval mortality when fed 1 mg/ml of *vgat* shRNA recombinant *Chlorella*; **c** larval mortality when fed 5 mg/ml of *vgat* shRNA recombinant *Chlorella*; **d** larval mortality when fed 15 mg/ml of *vgat* shRNA recombinant *Chlorella*; **e** larval mortality when fed 25 mg/ml of *vgat* shRNA recombinant *Chlorella*. ‘Feed’ refers to the larval diet fed to the larvae; ‘vgat 6,’ ‘vgat 15,’ and ‘vgat 22’ refer to larvae fed *vgat* shRNA recombinant *Chlorella* lines 6, 15, and 22, repectively. Significant differences (*P* < 0.05, Duncan’s multiple range test) are indicated by different letters

To determine the optimal lethal dose of *Ae. albopictus* larvae using shRNA recombinant *Chlorella*, a feeding trial was conducted over 15 days with 10 first instar larvae per group. Under identical feeding conditions, the mortality rates of larvae fed on the recombinant *Chlorella* lines vgat6, vgat15, and vgat22, at a concentration of 0.25 mg/ml, were 76.7%, 63.3%, and 93.3%, respectively. In comparison, larvae fed a larval diet at the same concentration had a mortality rate of 83.3%. At a concentration of 1 mg/ml, the mortality rates for larvae fed recombinant *Chlorella* lines vgat6, vgat15, and vgat22 were 43.3%, 40.0%, and 36.7%, respectively, while larvae fed a larval diet at the same concentration had a mortality rate of 36.7%. At a concentration of 5 mg/ml, the mortality rates for larvae fed recombinant *Chlorella* lines vgat6, vgat15, and vgat22 were 16.7%, 6.7%, and 46.7%,, respectively, while larvae fed a larval diet at the same concentration had a mortality rate of 16.7%. At a concentration of 15 mg/ml, the mortality rates for larvae fed recombinant *Chlorella* lines vgat6, vgat15, and vgat22 were 63.3%, 70.0%, and 63.3%, respectively, while larvae fed a larval diet at the same concentration had a 3.3% mortality rate. Lastly, at a concentration of 25 mg/ml, the mortality rates for larvae fed *Chlorella* lines vgat6, vgat15, and vgat22 were 56.7%, 60.0%, and 56.7%, respectively, with no mortality (0.0%) observed in larvae fed a larval diet at the same concentration (Fig. 4, Additional file 1: Fig. S3). Together, these results demonstrate that lower doses of *Chlorella* (or a larval diet) result in higher larval mortality due to food deprivation. Moreover, feeding shRNA recombinant *Chlorella* at a concentration of 15 mg/ml was optimally effective in inducing lethality compared with only 3.3% larval mortality using the control feed at the same concentration.

**Fig. 5:**
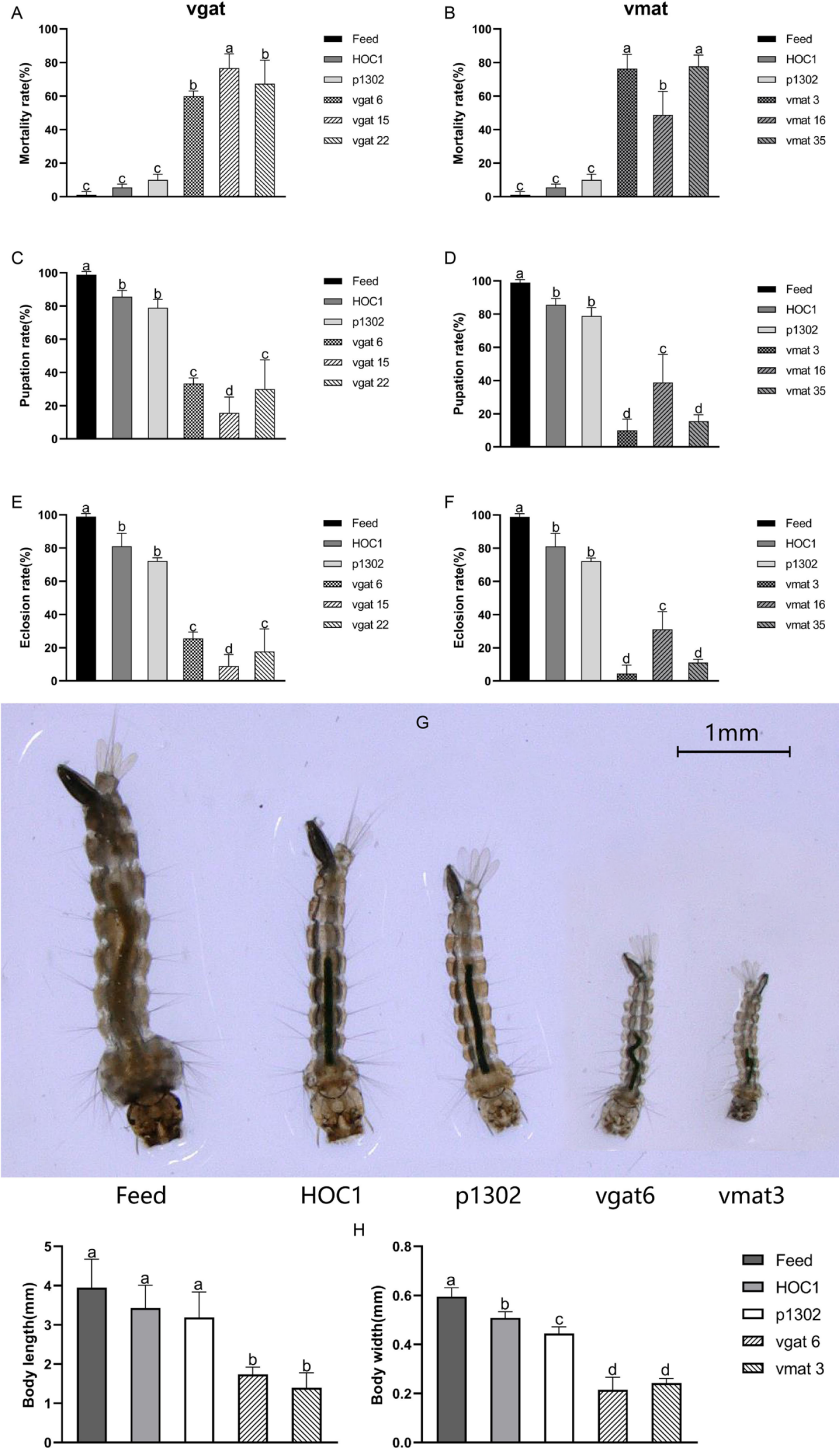
Mortality, pupation and emergence rates, and the length and width of larvae fed *vgat* or *vmat* shRNA recombinant *Chlorella*. **a** Larval mortality rate when fed *vgat* shRNA recombinant *Chlorella*; **b** larval mortality rate when fed *vmat* shRNA recombinant *Chlorella*; **c** larval pupation rate when fed *vgat* shRNA recombinant *Chlorella*; **d** larval pupation rate when fed *vmat* shRNA recombinant *Chlorella*; **e** larval eclosion rate when fed *vgat* shRNA recombinant *Chlorella*; **f** larval eclosion rate when fed *vmat* shRNA recombinant *Chlorella*; **g** and **h** length and width of L3 larvae from each treatment were measured. Feed, larval diet fed to larvae; HOC1, wild *Chlorella vulgaris* HOC1 fed larvae; p1302, larvae fed an empty pCAMBIA1302 plasmid from transgenic *Chlorella*; vgat6, vgat15, and vgat 22, larvae fed a *vgat* shRNA from recombinant *Chlorella* lines 6, 15, and 22; vmat3, vmat16, and vmat35, larvae fed a *vmat* shRNA from recombinant *Chlorella* lines 3, 16, and 35. For (H), significant differences (*P* < 0.05, Duncan’s multiple range tests) are shown by different letters

Using the optimal *Chlorella* concentration (15 mg/ml), new feeding trials were performed on groups of 30 first-instar larvae. Observations revealed that larvae fed a larval diet or *C. vulgaris* HOC1 began dying on day 4 and that larvae fed pCAMBIA1302 transgenic *Chlorella* began dying on day 3. In comparison, larvae fed *vgat* shRNA recombinant *Chlorella* (vgat6, vgat15, and vgat22) or *vmat* shRNA recombinant *Chlorella* (vmat3, vmat16, and vmat35) began dying on day 1. The mortality rates for larvae fed *vgat* shRNA recombinant *Chlorella* were 60.0%, 76.7%, and 67.3% for vgat6, vgat15, and vgat22, respectively. The mortality rates for larvae fed *vmat* shRNA recombinant *Chlorella* were 76.3%, 48.8%, and 77.7% for vmat3, vmat16, and vmat35, respectively (see Fig. 5A and B and Additional file 1: Fig. S4). These findings provide evidence that ingestion of *vgat* or *vmat* shRNA-recombinant *Chlorella* has a lethal effect on *Ae. albopictus* larvae.

Observations of larval pupation revealed that larvae fed a larval diet or *C. vulgaris* HOC1 began pupation on day 5 and that larvae fed pCAMBIA1302 transgenic *Chlorella* began pupation on day 6. The overall pupation rates for the three groups of larvae (those fed a larval diet, those fed *C. vulgaris* HOC1, and those fed pCAMBIA1302 transgenic *Chlorella*) were 98.9%, 85.5%, and 78.8%, respectively. In comparison, larvae fed *vgat* shRNA recombinant *Chlorella* (vgat6, vgat15, and vgat22) exhibited pupation rates of 33.3%, 8.9%, and 30.0%, respectively, while larvae fed *vmat* shRNA recombinant *Chlorella* (vmat3, vmat16, vmat35) exhibited pupation rates of 10.0%, 38.9%, and 15.5%, respectively (Fig. 5C and D, Additional file 1: Fig. S4).

Observations of adult emergence revealed that larvae fed a larval diet or *C. vulgaris* HOC1 began to emerge on day 6, while larvae fed pCAMBIA1302 transgenic *Chlorella* began to emerge on day 7. The overall adult emergence rates for the above three diets were 98.9%, 81.1%, and 72.2%, respectively. In comparison, larvae fed on *vgat* shRNA recombinant *Chlorella* (vgat6, vgat15, and vgat22) exhibited adult emergence rates of 28.9%, 6.7%, and 25.6%, respectively, while larvae fed on *vmat* shRNA recombinant *Chlorella* (vmat3, vmat16, and vmat35) exhibited adult emergence rates of 5.5%, 31.1%, and 12.2%, respectively (see Fig. 5E and F and Additional file 1: Fig. S4). Based on the above results, vgat6 and vmat3 shRNA recombinant *Chlorella* were selected for all subsequent feeding experiments.

In observations of larval length, the longest L3 body length was observed in mosquitoes fed the larval diet (3.878 mm), followed by those fed *C. vulgaris* HOC1 (3.629 mm) and those fed pCAMBIA1302 transgenic *Chlorella* (3.377 mm). In comparison, the body lengths of mosquitoes fed shRNA recombinant *Chlorella* were significantly shorter than those of the controls (mosquitoes fed the larval diet, *C. vulgaris* HOC1, or pCAMBIA1302 transgenic *Chlorella*) (see Fig. 5G and H). In observations of larval body width, mosquitoes fed the larval diet had the widest L3 larval body width (0.592 mm), followed by those fed *C. vulgaris* HOC1 (0.446 mm) and those fed pCAMBIA1302 transgenic *Chlorella* (0.422 mm). In comparison, the body widths of mosquitoes fed a recombinant *Chlorella* diet were significantly narrower than those of the control groups (see Fig. 5G and H).

**Fig. 6:**
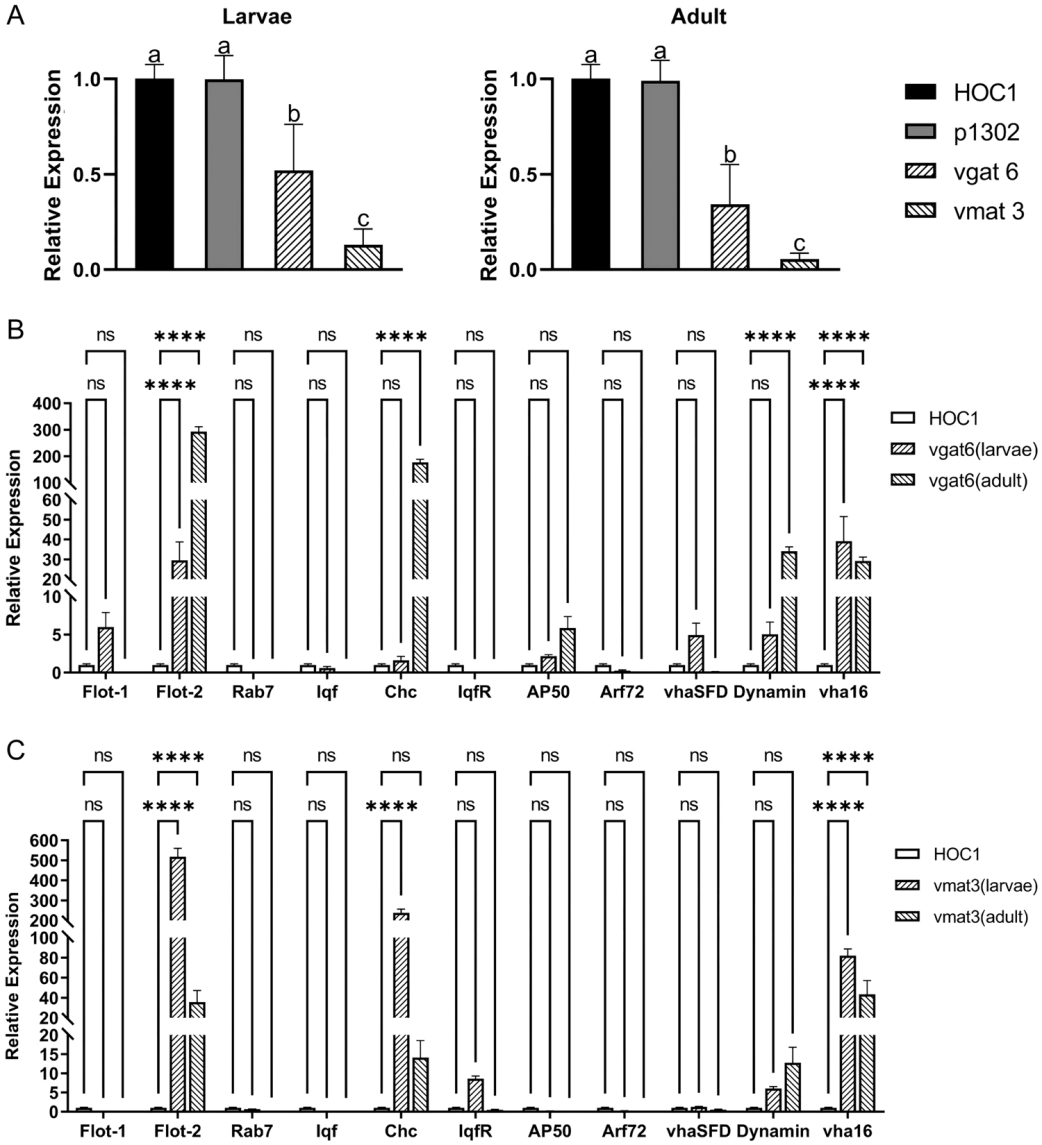
The mRNA levels of genes in *Aedes albopictus* L3 larvae or adults fed shRNA-recombinant *Chlorella*. **a** The relative *vgat and vmat* mRNA levels in *Ae. albopictus* L3 larvae or adults fed on shRNA recombinant *Chlorella*. **b** The relative *flot-1, flot2, Rab7, iqf, Chc, iqfR, AP50, Arf72, vhaSFD, Dynamin*, and *vha16* mRNA levels in *Ae. albopictus* L3 larvae or adults fed on *vgat* shRNA recombinant *Chlorella*. **c** The relative *flot-1, flot2, Rab7, iqf, Chc, iqfR, AP50, Arf72, vhaSFD, Dynamin*, and *vha16* mRNA levels in *Aedes albopictus* L3 larvae or adult fed on *vmat* shRNA recombinant *Chlorella*. HOC1, larvae or adults fed on wild *Chlorella vulgaris* HOC1; p1302, larvae or adults fed on the empty plasmid pCAMBIA1302 transgenic *Chlorella*; vgat6 and vmat3: larvae or adults fed on the shRNA recombinant *Chlorella* lines vgat6 and vmat3. Expression of CME related genes: clathrin adapter protein 50 (*AP50*), clathrin heavy chain (*Chc*), liquid facets (*lqf*), liquid facet-related protein (*lqfR*), *Dynamin*, vacuolar H + ATPase 16 kDa (*vha16*), V-type proton ATPase subunit H (*VhaSFD*), Ras-like GTPase (*Rab7*), ADP-ribosylation factor 72 (*Arf72*), flotillin-1 (*Flot-1*) and flotillin-2 (*Flot-2*). Results are expressed as the mean ± SE of three independent replicates. Asterisks (****) indicate statistically significant differences relative to controls (ANOVA with Dunnet's post hoc test, *p* < 0.0001), while ns indicates no significance. For **A**, significant differences (*P* < 0.05, Duncan’s multiple range test) are shown by different letters

Next, RT-qPCR was performed to evaluate the expression levels of the *vgat* and *vmat* genes in *Ae. albopictus* larvae (and the adults after maturation) fed shRNA recombinant *Chlorella*. Larvae fed on *C. vulgaris* HOC1 or pCAMBIA1302 transgenic *Chlorella* served as controls. In both larvae and adults, *vgat* and *vmat* mRNA expression levels in the groups fed shRNA recombinant *Chlorella* were significantly lower than *vgat* and *vmat* mRNA expression levels in the control groups (Fig. 6A). Additionally, the mRNA expression level of the *vmat* gene was markedly reduced compared to that of the *vgat* gene in both larvae and adults (12.9% *vs*. 52.0% in larvae, 5.5% *vs*. 34.2% in adults). Moreover, the most substantial reduction in gene expression was observed in adults fed on shRNA recombinant *Chlorella* line vmat3 (94.6%). Together, these results provide evidence that RNAi recombinant *Chlorella* can effectively silence the *vgat* and *vmat* genes in *Ae. albopictus*.

In addition, the levels of several gene transcripts involved in the clathrin-mediated endocytosis (CME) pathway were evaluated. These included coat assembly proteins (clathrin adapter* AP50*, clathrin heavy chain [*Chc*]), vesicle formation and budding proteins (liquid facets [*lqf*], liquid facet-related [*lqfR*], and *dynamin*), early endosome proteins (*Vha16*, *VhaSFD*), endosome maturation protein (*Rab7*), ADP-ribosylation factor-like 1 (*Arf72A*), and two genes of the flotillin-mediated endocytosis pathway (*flot-1*, *flot-2*). The RT-qPCR results revealed that the mRNA expression levels of *flot-1*, *flot-2*, *Chc*, *AP50*, *VhaSFD*, *Dynamin*, and *vha16* were significantly upregulated in larvae and adults fed on *vgat* shRNA recombinant *Chlorella* (Fig. 6B). Similarly, mRNA expression levels of *flot2*, *Chc*, *lqfR*, *Dynamin*, and *vha16* were significantly upregulated in larvae and adults fed on *vmat* shRNA recombinant *Chlorella* (Fig. 6C). Together, these results suggest that these genes may play a role in the RNAi response of *Ae. albopictus*.

### Attraction test evaluating various sources of sugar baits for *Ae. albopictus*

Mango, citrus, mulberry, dragon fruit, tomato, and poplar peach juices were tested for their attractiveness to adult *Ae. albopictus*. The results revealed that the mixture of mulberry juice and *Chlorella* was the most attractive to adult *Ae. albopictus* mosquitoes, provoking a biting rate of 23.4%. In second place was the mixture of citrus juice and *Chlorella*, with a biting rate of 18.3% (for comparison, the biting rate for the mixture of sucrose and *Chlorella* was 10.4%, while the biting rate for 10% sucrose solution was 7.2%). Hence, with the exception of dragon juice and tomato juice, mulberry juice, citrus juice, mango juice, and poplar peach juice were all more attractive to *Ae. albopictus* than sucrose solution (Table 1).

**Table 1:** Attraction test

Bait	Sucrose solution(10%)	Sucrose + *Chlorella*	Dragon fruit + *Chlorella*	Mango + *Chlorella*	Tomato + *Chlorella*	Citrus + *Chlorella*	Mulberry + *Chlorella*	Poplar peach + *Chlorella*
Bite number/30 min	6.67 ± 1.53	9.67 ± 1.53	6.67 ± 1.15	14.33 ± 2.52	5.67 ± 1.06	17.00 ± 3.61	21.67 ± 4.51	11.00 ± 3.61
Bite rate	7.2%	10.4%	7.1%	15.4%	6.1%	18.3%	23.4%	11.9%

*Bite rate* number of bites per treatment/total number of bites

### Attractive toxic sugar bait (ATSB) feeding trial and behavioral test of *Ae. albopictus*

**Fig. 7:**
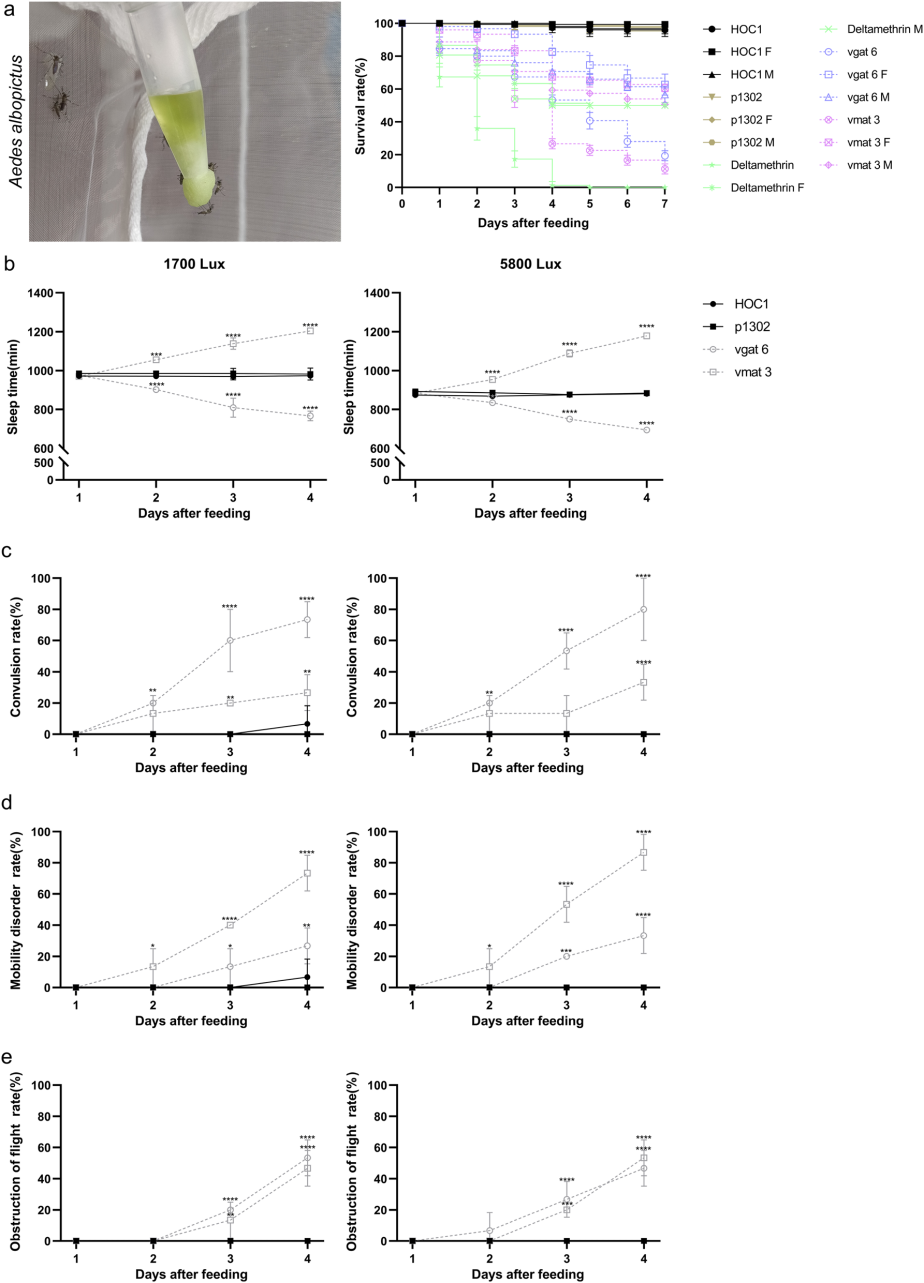
*Aedes albopictus* ATSB feeding trials and behavioral assays of adult *Ae. albopictus* fed on shRNA recombinant *Chlorella*. **a** Survival rates of *Ae. albopictus* after feeding on ATSB; **b** results of the mosquito sleep assay. Under normal light conditions (1700 lx), *Ae. albopictus* fed the *vgat* shRNA recombinant *Chlorella* had significantly reduced sleep times compared to the control group (*Ae. albopictus* fed *Chlorella vulgaris* HOC1) from the beginning of day 2 to day 4. In contrast, *Ae. albopictus* fed the *vmat* shRNA recombinant *Chlorella* had significantly increased sleep times compared to the control group. When daytime light intensity was increased to 5800 lx, sleeping time for the control group and for *Ae. albopictus* fed* vgat* shRNA recombinant *Chlorella* decreased, while sleep time for *Ae. albopictus* fed *vmat* shRNA recombinant *Chlorella* did not change significantly compared to *Ae. albopictus* at normal light intensity (day 4). This indicates that the increase in light intensity had less effect on *Ae. albopictus* fed *vmat* shRNA recombinant *Chlorella,* suggesting that these mosquitoes were insensitive to light enhancement. **c** Mosquitoes fed the *vgat* shRNA recombinant *Chlorella* twitched their legs considerably more than those in the control group. **d** The proportion of mosquitoes fed the *vmat* shRNA recombinant *Chlorella* that had impaired walking was substantially higher than in the control group. **e** When *vgat* and *vmat* shRNA recombinant *Chlorella* were fed to mosquitoes, the proportion with impaired flying was much higher than in the control group. *HOC1* organisms fed on wild *Chlorella vulgaris* HOC1 bait. *p1302* control, mosquitoes fed on pCAMBIA1302 transformed *Chlorella* bait. *vgat6* mosquitoes fed on *vgat* shRNA recombinant *Chlorella* line 6 bait. *vmat3* mosquitoes fed on *vmat* shRNA recombinant *Chlorella* line 3 bait. Deltamethrin: organisms fed on Deltamethrin bait. M refers to male and F to female. Asterisks (****) indicate statistically significant differences relative to the control group (ANOVA with Dunnet's post hoc test, *p* < 0.0001), while ns indicates no significance

An ATSB study was conducted over 7 days, starting 3 days after adult eclosion. As expected, many adult mosquitoes died within 24 h of consuming deltamethrin, with mortality reaching 100% on day 5 (50.0%, of females and 50.0% of males). Interestingly, adult mosquitoes fed shRNA recombinant *Chlorella* also experienced mass mortality within 24 h. The mortality rate of adult mosquitoes fed on the shRNA recombinant *Chlorella* line vgat6 was 80.7% (37.3% females, 43.4% males), while the mortality rate of adult mosquitoes fed on the shRNA recombinant *Chlorella* line vmat3 was 85.3% (38.6% females, 46.7% males). These mortality rates were significantly higher than the control mortality rate of 4.0% (females 0.6%, males 3.4%) for mosquitoes fed *C. vulgaris* HOC1 and 5.3% (females 2.6%, males 2.7%) for mosquitoes fed pCAMBIA1302 transgenic *Chlorella* (Fig. 7a).

The results of a 4-day sleep experiment, performed under normal light conditions (1,700 lx), revealed that mosquitoes fed on the *vgat* shRNA recombinant *Chlorella* toxin sugar bait experienced a gradual reduction in sleep duration (from 975 min on day 1 to 767 min on day 4). In comparison, the control group (fed *C. vulgaris* HOC1) exhibited stable sleep duration (day 1, 971 min; day 4, 973 min). The observed decrease in sleep time observed in the *vgat* shRNA group was statistically significant on days 2–4 (compared to the control group). Conversely, mosquitoes fed *vmat* shRNA recombinant *Chlorella* bait exhibited a significant increase in sleep duration (from 969 min on day 1 to 1204 min on day 4) compared to the control group (see Fig. 7b).

When the daytime light intensity was increased to 5800 lx, sleep duration significantly decreased in both the control (day 1, 874 min; day 4, 880 min) and the *vgat* shRNA group (day 1, 883 min; day 4, 693 min). However, sleep duration in the *vmat* shRNA group remained relatively stable (day 4: 1178 min), showing no significant difference compared to their sleep duration under normal light conditions (day 4: 1204 min). Hence, the *vmat* shRNA group is less sensitive to the increased light intensity (see Fig. 7b).

Additionally, behavioral analyses revealed that the *vgat* shRNA group displayed the highest prevalence of leg convulsions (see Fig. 7c). As shown in Fig. 7d, mosquitoes fed *vmat* shRNA recombinant *Chlorella* bait had the highest incidence of gait disorders. Furthermore, the incidences of flight disorders were significantly higher in the *vgat* and *vmat* shRNA groups compared with the control group (Fig. 7e). In summary, silencing the *vgat* gene in *Ae. albopictus* decreased sleep duration and caused leg twitching and impaired flight. Conversely, silencing the *vmat* gene increased sleep duration, leading to impaired locomotion, impaired flight, and a reduction in sensitivity to higher light intensity.

### ATSB tests on non-target organisms

**Fig. 8:**
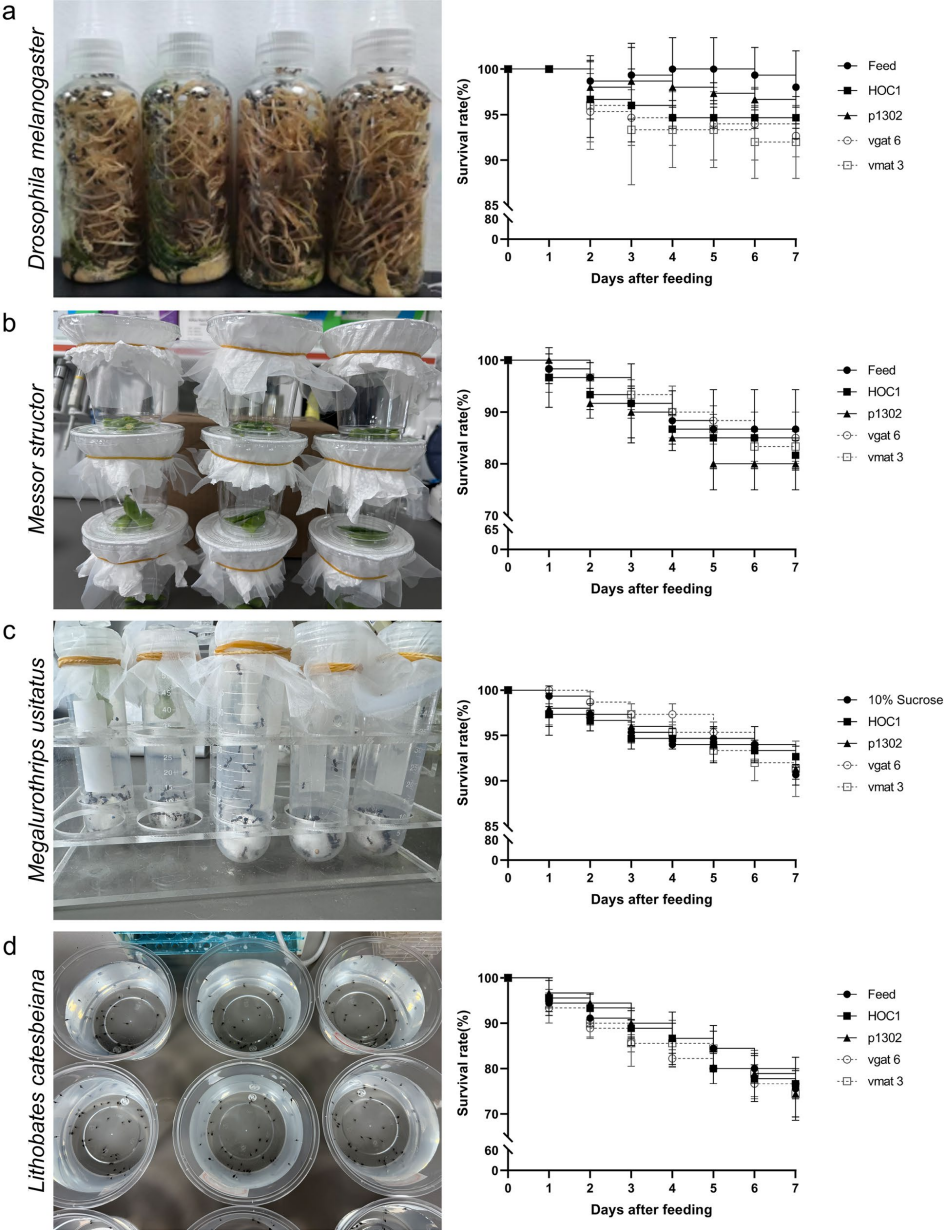
Non-target organism ATSB feeding trials Survival rates of* Drosophila melanogaster* **a**, *Megalurothrips usitatus* **b**, *Messor structor* **c**, and *Lithobates catesbeiana* tadpoles **d** after feeding on ATSB. ‘Feed’ refers to organisms fed on feed; ‘HOC1’ refers to organisms fed on wild *Chlorella vulgaris* HOC1 bait; ‘p1302’ refers to organisms fed on pCAMBIA1302-transformed *Chlorella* bait; ‘vgat6’ refers to organisms fed on *vgat*-shRNA recombinant *Chlorella* line 6 bait; ‘vmat3’ refers to organisms fed on *vmat*-shRNA recombinant *Chlorella* line3 bait

The results of additional tests conducted on several non-target organisms, *D. melanogaster*, *Messor structor*, *Megalurothrips usitatus*, and *Lithobates catesbeiana* tadpoles, are summarized below. The survival rate of *D. melanogaster* fed *C. vulgaris* HOC1 was 94.7%. The survival rate of *D. melanogaster* fed the pCAMBIA1302 transgenic *Chlorella* was 94.7%. The survival rate of *D. melanogaster* fed the larval diet was 96.0%. The survival rates of *D. melanogaster* fed *vgat* shRNA recombinant *Chlorella* and *vmat* shRNA recombinant *Chlorella* were 92.7% and 92.0%, respectively. A statistical analysis revealed that there was no significant difference in *D. melanogaster* survival rate between groups fed shRNA recombinant *Chlorella* and the control groups (*C. vulgaris* HOC1, pCAMBIA1302 transgenic *Chlorella*, and the larval diet) (Fig. 8a). Likewise, there was no significant difference in *M. usitatus*, *M. structor*, and *L. catesbeiana* tadpole survival rates in the shRNA recombinant *Chlorella* fed groups compared to those fed the controls (see Fig. 8b, c and d).

### *Aedes albopictus* semi-field trial

**Fig. 9:**
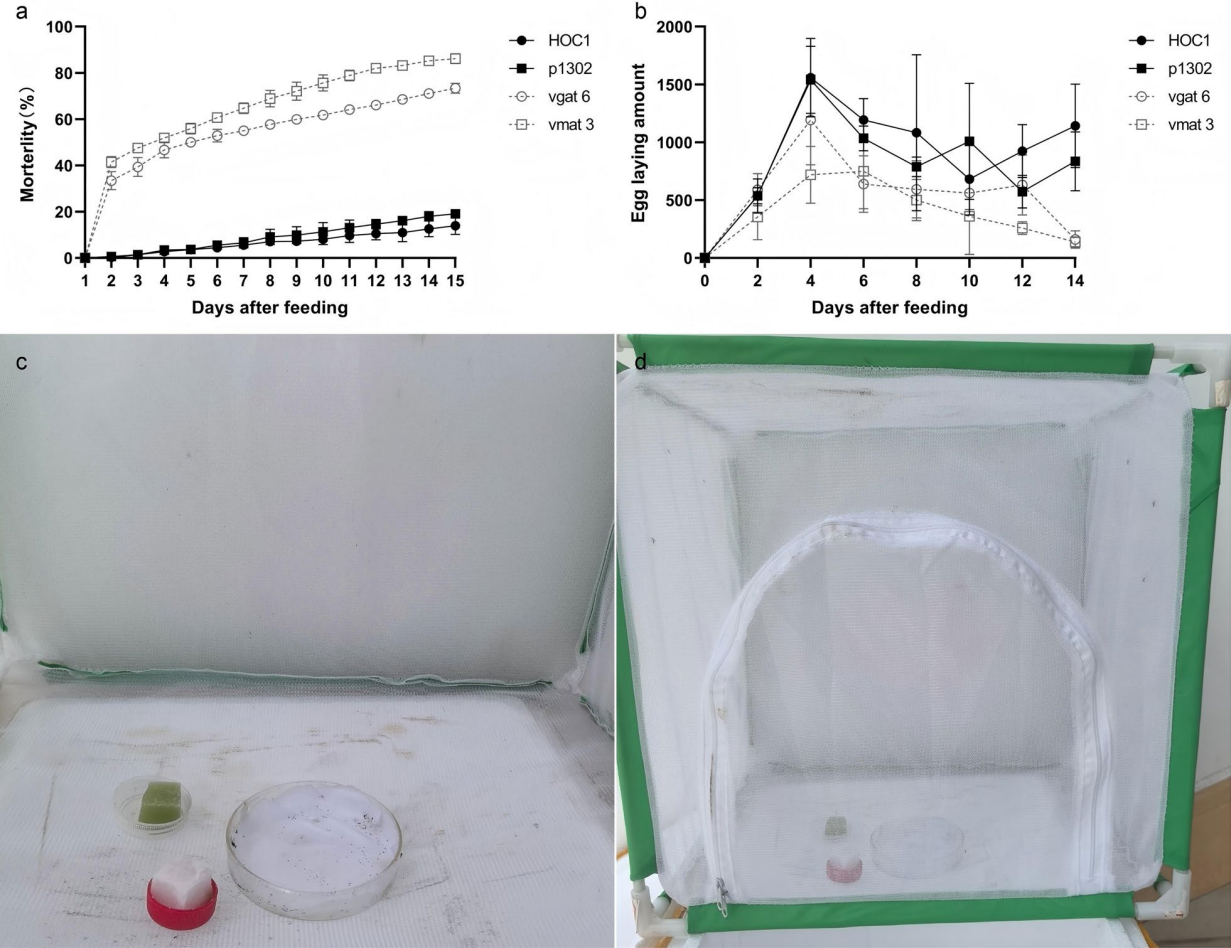
Semi-field trial. **a** Mortality of *Aedes albopictus* after feeding on ATSB. **b** Number of eggs laid by *Ae. albopictus* after feeding on ATSB. **c** and **d** Semi-field test setup. HOC1, control, organisms fed on wild *Chlorella vulgaris* HOC1 bait. p1302, control, mosquitoes fed on pCAMBIA1302 transformed *Chlorella* bait. vgat 6, mosquitoes fed on *vgat* shRNA recombinant *Chlorella* line 6 bait. vmat3, mosquitoes fed on *vmat* shRNA recombinant *Chlorella* line 3 bait

A semi-field trial revealed that many adult mosquitoes administered the ATSBs died within 24 h, although the mortality rate decreased after 48 h. The overall mortality rate increased slowly. The mosquito mortality rates were as follows: *C. vulgaris* HOC1 containing ATSB, 13.9%; pCAMBIA1302 transgenic *Chlorella* containing ATSB, 19.1%; *vgat* shRNA recombinant *Chlorella* containing ATSB, 73.3%; *vmat* shRNA recombinant *Chlorella* containing ATSB, 86.1%. Hence, the ATSB prepared with *vgat* shRNA recombinant *Chlorella* and *vmat* shRNA recombinant *Chlorella* demonstrated high toxicity to *Ae. albopictus* (see Fig. 9a, c and d). In addition, changes in mosquito egg production were observed after ATSB administration (Fig. 9b, Additional file 1: Fig. S5). The total numbers of eggs laid by mosquitoes in each group were as follows: *C. vulgaris* HOC1 containing ATSB, 7668; pCAMBIA1302 transgenic *Chlorella* containing ATSB, 6316; *vgat* shRNA recombinant *Chlorella* containing ATSB, 4366; *vmat* shRNA recombinant *Chlorella* containing ATSB, 3081. Hence, mosquitoes fed shRNA recombinant *Chlorella* ATSB laid considerably fewer eggs than the control groups (those fed *C. vulgaris* HOC1 or pCAMBIA1302 transgenic *Chlorella*).

## Discussion

RNAi is an endogenous regulatory pathway in eukaryotic cells that silences gene expression by generating small interfering RNAs (siRNAs). While this technology is already established as a useful research tool, it has more recently attracted the interest of the agricultural pest control community because of its potential applications in controlling agricultural pests and vector insects [72, 73]. However, RNAi efficiency has been observed to vary significantly between insect species. In beetles (Tenebrionidae, Chrysomelidae, and other families), for example, RNAi is efficient and systemic [74–76]. An injection of dsRNA is also able to induce efficient RNAi in hemipteran insects such as *Oncopeltus fasciatus*, *Halyomorpha halys*, and *Cimex lectularius*, as well as other insects such as *Blattella germanica*, *Schistocerca gregaria*, and *Locusta migratoria* [77–81]. In contrast, RNAi is inefficient in Lepidoptera [82]. In med fly (*Ceratitis capitata*), injection is a more effective method of delivering dsRNA than feeding [83]. Fortunately, reports on the use of RNAi insecticides for controlling mosquito populations are promising. To date, several siRNAs targeting essential mosquito genes have been reported to kill mosquito larvae and adults under laboratory conditions. Moreover, short hairpin RNAs (shRNAs) corresponding to a siRNA sequence have been transformed into *Saccharomyces cerevisiae* and used to kill *Aedes*, *Anopheles*, and *Culex* mosquitoes [84–87]. In addition, dsRNAs corresponding to key *Anopheles gambiae* genes have been successfully expressed in *Escherichia coli*. Mosquitoes fed heat-inactivated strains exhibited defective salivary gland morphology [88], providing evidence that silencing target genes through oral feeding could be a means of studying functionally important genes in mosquitoes.

ATSB is an emerging vector control method that exploits the sugar-feeding behavior of mosquitoes to attract them to a source of sugar containing toxins, thereby controlling and reducing mosquito populations [89–91]. Although sugar baits reduce pesticide use, pesticide resistance remains a concern [92]. In addition, many of the low-toxicity insecticides currently used, such as garlic oil and boric acid insecticides, are not mosquito specific, and long-term use may reduce the number of non-target organisms and cause an unwanted ecological impact [90]. Hence, increasing the specificity of pesticides is important for environmental safety, and it will further promote the development and widespread application of ATSB technology.

In the present study, the lethality of recombinant *Chlorella* expressing *vgat* or *vmat* shRNA against *Ae. albopictus* larvae was investigated. The results revealed that the lethality of *vgat* shRNA recombinant *Chlorella* ranged from 60.0% to 76.7%, while the lethality of *vmat* shRNA recombinant *Chlorella* ranged from 48.8% to 77.7% (Fig. 6a and b). Moreover, silencing of the *vgat* and *vmat* genes leads to the death of mosquito larvae and a significant reduction in their body length and width. Since mosquito larvae inhabit aquatic environments, recombinant *Chlorella* must be introduced into water bodies at sufficient concentrations to exert lethal effects on larvae [22, 23, 25]. However, environmental release of recombinant *Chlorella* into natural water systems (e.g. lakes and reservoirs) raises significant biosafety and ecological concerns because of risks of horizontal gene transfer to native species. Regulatory approval for such releases is typically protracted and challenging to secure within short time frames. To circumvent these limitations, an alternative strategy was developed in this study, with ATSBs incorporating shRNA-expressing recombinant *Chlorella* being deployed to target adult mosquitoes.

According to the results, the shRNA recombinant *Chlorella* vgat6 ATSB was 80.7% lethal to adult mosquitoes (37.3% in females and 43.4% in males), while the shRNA recombinant *Chlorella* vmat3 ATSB was 85.3% lethal to adult mosquitoes (38.6% in females and 46.7% in males) (Fig. 7a). Hence, shRNA recombinant *Chlorella* baits were effective in killing mosquitoes. An attractiveness test of different sugar source baits to *Ae. albopictus* revealed that mulberry juice was the most attractive to *Ae. albopictus*, followed by citrus juice (Table 1). These natural products were purchased from local supermarkets and should be considered a viable alternative to sucrose solution for the preparation of ATSB. In non-target organism toxicity tests, recombinant *Chlorella* were not lethal to *D. melanogaster*, *M. usitatus*, *M. structor*, or *L. catesbeiana* tadpoles (Fig. 8). These results demonstrate the specificity of the shRNA recombinant *Chlorella* ATSB mosquito killer and its biosafety. The results of our semi-field trial provided further evidence that *vgat* shRNA recombinant *Chlorella* ATSB and *vmat* shRNA recombinant *Chlorella* ATSB exhibited higher lethality against *Ae. albopictus* (73.3% and 86.1%, respectively) and caused a significant reduction in the numbers of their eggs laid (Fig. 9).

The *Ae. albopictus* genome contains only one *vgat* gene and one *vmat* gene. Similarly, the *D. melanogaster* genome contains a *dvgat* gene and a *dvmat* gene [93]. The *dvgat* gene is expressed in the GABAergic neurons of both larval and adult *D. melanogaster*, and mutations in this gene are lethal [94, 95]. In flies, the GABAergic neurons are known to promote sleep. The effects of GABA are mediated in part through the Rdl (Resistant to dieldrin) GABA receptor, which promotes sleep and is especially important in regulating sleep latency [95–98]. Dorsal paired median GABAergic neurons, which target the MBs (mushroom bodies) to promote sleep, provide at least one site of action [99]. Furthermore, mutations in the glial GABA-metabolizing enzyme, GABA transaminase, are known to increase sleep, presumably by increasing GABA levels [100], providing a potential link between GABA and sleep homeostasis in *D. melanogaster*.

The gene encoding *D. melanogaster dvmat* has two splice variants, *dvmat*-A and *dvmat*-B [101]. *dvmat*-A is expressed in all dopaminergic, serotonergic, and octopaminergic cells in both larvae and adults [101, 102]. A method involving the introduction of mutations in the *dvmat* gene and selective rescue of *dvmat* mutant strains in single or multiple aminergic systems was used to determine its function in dopaminergic, 5-HTergic, octopaminergic, and tyrosinergic neurons [103]. According to the results, dopamine plays an important role in behaviors such as sleep, arousal, photoreception, circadian rhythms, and feeding [104–112]; 5-HT plays a role in aggression, positional memory, circadian rhythms, and sleep [113–115]; octopamine plays an important role in *D. melanogaster* sleep, locomotion, female fertility, aggression, environmental memory, and response to environmental stressors [116–123]. Additional studies have demonstrated the existence of compensation and substitution mechanisms between these aminergic systems [124].

The results of our *Ae. albopictus* behavioral assay revealed that sleep time was reduced in mosquitoes fed *vgat* shRNA recombinant *Chlorella* ATSB. Thus, feeding induced a knockdown of the mosquito *vgat* gene, and this consequently reduced mosquito sleep time, which is consistent with the known function of VGAT in GABAergic neurons (Fig. 7b) [95–98]. In addition, knockdown of the *vgat* gene induced leg twitching and impaired flight in mosquitoes. Conversely, sleep time was increased in mosquitoes fed *vmat* shRNA recombinant *Chlorella* ATSB (Fig. 7b, c, d, and e). In addition, knockdown of the *vmat* gene caused walking and flight problems in mosquitoes and modulated their sensitivity to light (Fig. 7b, c, d, and e). These observations are consistent with the known roles of VMAT-transmitted dopamine, 5-HT, and octopamine in regulating insect sleep and light sensitivity [111–113, 120]. Similarly, Hapairai et al. (2020) observed flight and locomotor deficits in *Ae. aegypti* after interference of the *dop1* (dopamine receptor 1) gene in mosquito dopaminergic neurons [68].

While nematodes use SID (systemic RNA interference-defective) to transport dsRNA into the cell and to transport dsRNA between cells [125], insects mainly use clathrin-mediated endocytosis (CME) to take up dsRNA [126, 127]. In the present study, we analyzed the mRNA expression levels of several important genes involved in the CME pathway before and after knockdown of the *vgat* or *vmat* genes. The results revealed that mRNA expression levels of *flot-1*, *flot2*, *Chc*, *AP50*, *VhaSFD*, *dynamin*, and *vha16* were significantly upregulated after *vgat* was knocked down (Fig. 4B). Similarly, mRNA expression levels of *flot2*, *Chc*, *lqfr*, *dynamin*, and *vha16* were significantly upregulated after *vmat* was knocked down (Fig. 4C). Together, these results suggest that *vgat* or *vmat* shRNAs may be taken up through CME in mosquito intestinal epithelial cells, an observation that complements the work of Abbasi et al. (2020) in *Ae. aegypti* [128].

## Conclusions

In the present study, *vgat* and *vmat* genes in the neurotransmitter transduction pathway of *Ae. albopictus* were selected as RNAi targets, and the shRNA recombinant *Chlorella* obtained after genetic transformation exhibited high lethality, specificity, and safety against both larvae and adult *Ae. albopictus*. The results of this study are a significant contribution to research on the use of recombinant microalgae as a biocide against mosquitoes. Moreover, knockdown of mosquito target genes by oral administration of shRNA provides a convenient way to test mosquito gene function.

## Supplementary Information

**Additional file 1: Table S1.** Primers used in this study. **Table S2.** *Ae. albopictus* endocytic genes and related sequences. **Table S3.** The origin, taxonomy, and Genbank accession number of the VGAT orthologs used in the present study, along with their homology rates with shRNA. **Table S4.** The origin, taxonomy, and Genbank accession number of the VMAT orthologs used in this study, along with their homology rates with shRNA. **Fig. S1.** Sequence of *vgat* and *vmat* shRNA and predicted shRNA secondary structure. **Fig. S2.** PCR electrophoresis results of recombinant plasmid pCAMvgat-shRNA (A) and pCAMvmat-shRNA (B) transgenic algal lines. **Fig. S3.** Mortality of mosquito larvae fed with *vgat* shRNA recombinant *Chlorella.* **Fig. S4.** Mortality, pupation, and emergence rates of larvae fed *vgat* or *vmat* shRNA recombinant *Chlorella.* **Fig. S5.** Paper with eggs laid by *Ae. albopictus* in semi-field trial.

## Data Availability

The data sets supporting the conclusions are available in this article and supplementary data.
