# Supplementary material for: Control of Aedes albopictus populations by silencing of the vesicular GABA transporter (vgat) and the vesicular monoamine transporter (vmat) genes using recombinant Chlorella shRNA
Source: Parasit Vectors. 2025 Oct 14;18:414. doi: 10.1186/s13071-025-07053-x (PMC12522392; doi:10.1186/s13071-025-07053-x)
Supplement: Supplementary file 1 — Additional file 1: Table S1. Primers used in this study. Table S2. Ae. albopictus endocytic genes and related sequences. Table S3. The origin, taxonomy, and Genbank accession number of the VGAT orthologs used in the present study, along with their homology rates with shRNA. Table S4. The origin, taxonomy, and Genbank accession number of the VMAT orthologs used in this study, along with their homology rates with shRNA. Fig. S1. Sequence of vgat and vmat shRNA and predicted shRNA secondary structure. Fig. S2. PCR electrophoresis results of recombinant plasmid pCAMvgat-shRNA (A) and pCAMvmat-shRNA (B) transgenic algal lines. Fig. S3. Mortality of mosquito larvae fed with vgat shRNA recombinant Chlorella. Fig. S4. Mortality, pupation, and emergence rates of larvae fed vgat or vmat shRNA recombinant Chlorella. Fig. S5. Paper with eggs laid by Ae. albopictus in semi-field trial. [file 13071_2025_7053_MOESM1_ESM.docx]

Additional file 1:

Table S1. Primers used in this study

| Genes | Primer name | Sequence (5'→3') | Gene ID (Vectorbase /Gene bank) |
| --- | --- | --- | --- |
| *vgat* | *vgat*-F | CCGTTTACCAACCCGAAAG | AALF004203 |
|  | *vgat*-R | TGATTGAACACTGCCTTGC |  |
| *vmat* | *vmat*-F | TCCTGTTCGTCCGTGTCC | AALFPA057308 |
|  | *vmat*-R | AACCACCTAATGCTATGC |  |
| *AP50* | *AP50-F* | ATCAAGTATCGCCGCAAT | AALC636_033072 |
|  | *AP50-R* | ACGCACCTCAATCTTCTG |  |
| *Chc* | *Chc-F* | AGCAGTGGATGTCTTCTTC | AALC636_022815 |
|  | *Chc-R* | GCATTAGCAGCAACCTTG |  |
| *lqf* | *lqf-F* | CAGTGAAGAGTATGGCAGAT | [AALC636_033617](https://vectorbase.org/vectorbase/app/record/gene/AALC636_033617) |
|  | *lqf-R* | AGAAGCAGGAATGGAGTTG |  |
| *lqfR* | lqfR-F | CAGTGAAGAGTATGGCAGAT | AALFPA_060726 |
|  | lqfR-R | AGAAGCAGGAATGGAGTTG |  |
| *Dynamin* | *Dynamin-F* | TCAATCAGCAACTCACCAA | AALFPA_067314 |
|  | *Dynamin-R* | TGTATTCCTCCACCTCCTT |  |
| *Vha16* | *Vha16-F* | GCGTTAGGAGCAGCATAT | AALC636_000071 |
|  | *Vha16-R* | TGTATTCCTCCACCTCCTT |  |
| *VhaSFD* | *VhaSFD-F* | GAACCTTGAGCAGCAGAA | AALFPA_048766 |
|  | *VhaSFD-R* | ATCATCAGACACTTCCAACA |  |
| *Rab7* | *Rab7-F* | GGAAGACCTCGTTGATGAA | AALC636_020391 |
|  | *Rab7-R* | CTGTCGTCCACCATTACTT |  |
| *Arf72* | *Arf72-F* | CAGACGCAGTCATCTATGT | AALFPA_076650 |
|  | *Arf72-R* | ATCCACCAACCACTCCAT |  |
| *Flot-1* | *Flot-1-F* | TTGTCTTGCCGTGTATTCA | AALFPA_080924 |
|  | *Flot-1-R* | CTCCAGTGTCTCCAATGC |  |
| *Flot-2* | *Flot-2-F* | GGTGAAGGTGATGACTGAA | AALC636_000218 |
|  | *Flot-2-R* | GTTAGCGTGCCAAGGATA |  |
| *rps17* | *rps17*-F | AAGAAGTGGCCATCATTCCA | AAEL004175 |
|  | *rps17*-R | GGTCTCCGGGTCGACTTC |  |
| *pCAMBIA1302* | *1302-F* | TTACCCAACTTAATCGCCTTGCAG | AF234298 |
|  | *1302-R* | TATCGCAATGATGGCATTTGTAGG |  |

Table S2. *Aedes albopictus* endocytic genes and related sequences

| *Ae. albopictus* | | Orthologues | | | |
| --- | --- | --- | --- | --- | --- |
| Gene | Gene ID | *Ae. aegypti* | % Identity | *D. melanogaster* | % Identity |
| *AP50* | AALC636_033072 | AAEL003106 | 88.85 | CG7057 | 83.18 |
| *Chc* | AALC636_022815 | AAEL022819 | 89.46 | CG9012 | 76.76 |
| *lqf* | [AALC636_033617](https://vectorbase.org/vectorbase/app/record/gene/AALC636_033617) | AAEL009088 | 91.30 | CG8532 | 73.35 |
| *lqfR* | AALFPA_060726 | AAEL013724 | 84.20 | CG42250 | 80.58 |
| *Dynamin* | AALFPA_067314 | AAEL007288 | 89.69 | CG18102 | 73.61 |
| *Vha16* | AALC636_000071 | AAEL000291 | 85.29 | CG3161 | 82.35 |
| *VhaSFD* | AALFPA_048766 | AAEL006516 | 85.27 | CG17332 | 77.08 |
| *Rab7* | AALC636_020391 | AAEL003503 | 84.14 | CG5915 | 76.44 |
| *Arf72* | AALFPA_076650 | AAEL014177 | 81.90 | CG6025 | 80.80 |
| *Flot-1* | AALFPA_080924 | AAEL025017 | 88.88 | CG8200 | 78.49 |
| *Flot-2* | AALC636_000218 | AAEL004041 | 88.16 | CG32593 | 78.25 |

Table S3. The origin, taxonomy and Genbank accession number of the VGAT orthologues used in the present study, along with their homology rates with shRNA.

| Species | Phylum | Class | Gene ID | Note | shRNA homology rate |
| --- | --- | --- | --- | --- | --- |
| *Aedes albopictus* | Arthropoda | Insecta | NC_085138.1 |  | 100.00% |
| *Aedes aegypti* | Arthropoda | Insecta | NC_035109.1 |  | 100.00% |
| *Culex quinquefasciatus* | Arthropoda | Insecta | NC_068938.1 |  | 82.35% |
| *Anopheles darlingi* | Arthropoda | Insecta | NC_064875.1 |  | 95.65% |
| *Drosophila melanogaster* | Arthropoda | Insecta | NT_033778.4 |  | 66.67% |
| *Schistocerca americana* | Arthropoda | Insecta | NC_060124.1 |  | 81.82% |
| *Apis cerana* | Arthropoda | Insecta | NC_083859.1 |  | 75.00% |
| *Solenopsis invicta* | Arthropoda | Insecta | NC_052677.1 |  | 77.50% |
| *Dermacentor silvarum* | Arthropoda | Arachnida | NC_051163.1 |  | 64.00% |
| *Stegodyphus dumicola* | Arthropoda | Arachnida | NW_023316379.1 |  | 70.83% |
| *Penaeus chinensis* | Arthropoda | Malacostraca | NC_061841.1 |  | 76.00% |
| *Limulus polyphemus* | Arthropoda | Merostomata | NW_013670195.1 |  | 60.00% |
| *Xenopus laevis* | Chordata | Amphibia | NC_054388.1 |  | 69.57% |
| *Ambystoma mexicanum* | Chordata | Amphibia | XM_069652365.1 |  | 56.52% |
| *Electrophorus electricus* | Chordata | Actinopterygii | XM_027010731.2 |  | 61.90% |
| *Petromyzon marinus* | Chordata | Agnatha | XM_032952818.1 |  | 47.82% |
| *Meleagris gallopavo* | Chordata | Aves | XM_010722328.2 |  | 69.56% |
| *Scyliorhinus canicula* | Chordata | Chondrichthyes | XM_038803437.1 |  | 70.83% |
| *Branchiostoma belcheri* | Chordata | Leptocardii | XM_019768633.1 |  | 63.64% |
| *Homo sapiens* | Chordata | Mammalia | NC_000020.11 |  | 63.64% |
| *Macaca fascicularis* | Chordata | Mammalia | NC_088384.1 |  | 72.22% |
| *Mus musculus* | Chordata | Mammalia | NC_000068.8 |  | 72.22% |
| *Caenorhabditis elegans* | Nematoda | Secernentea | NC_003281.10 | Nuc-47/VGAT | 69.23% |
| *Anneissia japonica* | Echinodermata | Crinoidea | XM_033256539.1 |  | 65.22% |
| *Pomacea canaliculata* | Mollusca | Gastropoda | XM_025254040.1 |  | 65.00% |
| *Clytia hemisphaerica* | Cnidaria | Hydrozoa | [XP_066917743.1](https://www.ncbi.nlm.nih.gov/protein/XP_066917743.1?report=genbank&log$=prottop&blast_rank=1&RID=T0W1M9UA013) |  | 56.52% |
| *Exaiptasia diaphana* | Cnidaria | Hexacorallia | KXJ12303.1 |  | 72.00% |
| *Dimorphilus gyrociliatus* | Annelida | Polychaeta | [CAD5124019.1](https://www.ncbi.nlm.nih.gov/protein/CAD5124019.1?report=genbank&log$=prottop&blast_rank=1&RID=T0W2F38S016) |  | 66.67% |
| *Paralvinella palmiformis* | Annelida | Polychaeta | JAODUP010000920.1 |  | <40% |
| *Schmidtea mediterranea* | Platyhelminthes | Turbellaria | [AKN21635.1](https://www.ncbi.nlm.nih.gov/protein/AKN21635.1?report=genbank&log$=prottop&blast_rank=1&RID=T0W353FS013) |  | 69.56% |
| *Cryptocotyle lingua* | Platyhelminthes | Trematoda | [QQY02486.1](https://www.ncbi.nlm.nih.gov/protein/QQY02486.1?report=genbank&log$=prottop&blast_rank=1&RID=T0W3XT5A013) |  | 69.56% |
| *Oopsacas minuta* | Porifera | Hexactinellida | [KAI6659301.1](https://www.ncbi.nlm.nih.gov/protein/KAI6659301.1?report=genbank&log$=prottop&blast_rank=1&RID=T0W7RE1G016) |  | 77.27% |
| *Rotaria magnacalcarata* | Rotifera | Eurotatoria | [CAF1040261.1](https://www.ncbi.nlm.nih.gov/protein/CAF1040261.1?report=genbank&log$=prottop&blast_rank=1&RID=T0WAE6CX013) |  | 52.17% |
| *Brachionus calyciflorus* | Rotifera | Monogononta | [CAF0948778.1](https://www.ncbi.nlm.nih.gov/protein/CAF0948778.1?report=genbank&log$=prottop&blast_rank=2&RID=XJEATCRS016) |  | 60.86% |

Table S4. The origin, taxonomy and Genbank accession number of the VMAT orthologues used in this study, along with their homology rates with shRNA.

| Species | Phylum | Class | Gene ID | Note | shRNA homology rate |
| --- | --- | --- | --- | --- | --- |
| *Aedes albopictus* | Arthropoda | Insecta | XP_062714849.1 |  | 100.00% |
| *Aedes aegypti* | Arthropoda | Insecta | [XP_021705603.1](https://www.ncbi.nlm.nih.gov/protein/XP_021705603.1?report=genbank&log$=prottop&blast_rank=1&RID=T16X85FT013) |  | 100.00% |
| *Culex quinquefasciatus* | Arthropoda | Insecta | XP_038118028.1 |  | 90.48% |
| *Anopheles coluzzii* | Arthropoda | Insecta | [XP_040222518.1](https://www.ncbi.nlm.nih.gov/protein/XP_040222518.1?report=genbank&log$=prottop&blast_rank=1&RID=T173741K016) |  | 52.38% |
| *Aphis gossypii* | Arthropoda | Insecta | [XP_027847742.1](https://www.ncbi.nlm.nih.gov/protein/XP_027847742.1?report=genbank&log$=prottop&blast_rank=1&RID=T16A8ZWX016) |  | 66.67% |
| *Bradysia coprophila* | Arthropoda | Insecta | [XP_037032911.1](https://www.ncbi.nlm.nih.gov/protein/XP_037032911.1?report=genbank&log$=prottop&blast_rank=1&RID=T16DJ95V013) |  | 64.29% |
| *Drosophila melanogaster* | Arthropoda | Insecta | [NP_001014524.1](https://www.ncbi.nlm.nih.gov/protein/NP_001014524.1?report=genbank&log$=prottop&blast_rank=1&RID=T16EC18M016) |  | 68.18% |
| *Solenopsis invicta* | Arthropoda | Insecta | [XP_011166113.1](https://www.ncbi.nlm.nih.gov/protein/XP_011166113.1?report=genbank&log$=prottop&blast_rank=1&RID=T16HT6V8016) |  | 66.67% |
| *Cherax quadricarinatus* | Arthropoda | Malacostrac | [XP_069935801.1](https://www.ncbi.nlm.nih.gov/protein/XP_069935801.1?report=genbank&log$=prottop&blast_rank=1&RID=T16KBBJZ016) |  | 71.43% |
| *Penaeus chinensis* | Arthropoda | Malacostrac | [XP_047478437.1](https://www.ncbi.nlm.nih.gov/protein/XP_047478437.1?report=genbank&log$=prottop&blast_rank=1&RID=T16MT60P016) |  | 76.19% |
| *Dermacentor albipictus* | Arthropoda | Arachnida | XP_065295791.1 |  | 57.14% |
| *Helobdella robusta* | Annelida | Hirudinea | XM_009022091.1 |  | 71.43% |
| *Capitella teleta* | Annelida | Polychaeta | [KB302824.1](https://www.ncbi.nlm.nih.gov/nuccore/KB302824.1) |  | 61.90% |
| *Ridgeia piscesae* | Annelida | Polychaeta | KAK2168852.1 |  | 42.86% |
| *Stylophora pistillata* | Cnidaria | Hexacorallia | XP_022783706.1 |  | 66.67% |
| *Montipora foliosa* | Cnidaria | Anthozoa | XP_068709519.1 |  | 66.67% |
| *Orbicella faveolata* | Cnidaria | Anthozoa | XM_020768078.1 |  | 52.38% |
| *Geotrypetes seraphini* | Chordata | Amphibia | XP_033795988.1 |  | 71.43% |
| *Catharus ustulatus* | Chordata | Aves | XP_032922371.1 |  | 57.14% |
| *Myxine glutinosa* | Chordata | Cyclostomata | [XP_067967837.1](https://www.ncbi.nlm.nih.gov/protein/XP_067967837.1?report=genbank&log$=prottop&blast_rank=1&RID=T16P5TH1013) |  | 61.90% |
| *Hemiscyllium ocellatum* | Chordata | Chondrichthyes | XP_060698110.1 |  | 66.67% |
| *Homo sapiens* | Chordata | Mammalia | [JAAKGM020000002.1](https://www.ncbi.nlm.nih.gov/nuccore/JAAKGM020000002.1) | VMAT2 | 57.14% |
| *Mus musculus* | Chordata | Mammalia | [XP_011240564.1](https://www.ncbi.nlm.nih.gov/protein/XP_011240564.1?report=genbank&log$=prottop&blast_rank=1&RID=T16SVD4Z013) |  | 42.86% |
| *Conger conger* | Chordata | Osteichthyes | XP_061084427.1 |  | 66.67% |
| *Emydura macquarii* | Chordata | Reptilia | XP_067417386.1 |  | 61.90% |
| *Patiria miniata* | Echinodermata | Asteroidea | XM_038215104 |  | 71.43% |
| *Acanthaster planci* | Echinodermata | Asteroidea | XM_022245883.1 |  | 69.57% |
| *Ostrea edulis* | Mollusca | Bivalvia | [XP_048746822.2](https://www.ncbi.nlm.nih.gov/protein/XP_048746822.2?report=genbank&log$=prottop&blast_rank=1&RID=T16UAS7A013) |  | 66.67% |
| *Mizuhopecten yessoensis* | Mollusca | Bivalvia | [XP_021371911.1](https://www.ncbi.nlm.nih.gov/protein/XP_021371911.1?report=genbank&log$=prottop&blast_rank=1&RID=T16UTKVD016) |  | 66.67% |
| *Pomacea canaliculata* | Mollusca | Gastropoda | XM_025254040.1 |  | 42.85% |
| *Caenorhabditis elegans* | Nematoda | Chromadorea | NC_003284.9 |  | 42.85% |
| *Schmidtea mediterranea* | Platyhelminthes | Turbellaria | [AGS83413.1](https://www.ncbi.nlm.nih.gov/protein/AGS83413.1?report=genbank&log$=prottop&blast_rank=7&RID=T0JESK04013) |  | 50% |
| *Opisthorchis felineus* | Platyhelminthes | Trematoda | TGZ58523.1 |  | 61.90% |
| *Oscarella lobularis* | Porifera | Homoscleromorpha | XP_065840153.1 |  | 52.38% |
| *Sycon ciliatum* | Porifera | Calcarea | XP_065182825.1 |  | 47.62% |
| *Rotaria socialis* | Rotifera | Eurotatoria | CAF3176309.1 |  | 47.62% |
| *Brachionus calyciflorus* | Rotifera | Monogononta | [CAF0807189.1](https://www.ncbi.nlm.nih.gov/protein/CAF0807189.1?report=genbank&log$=prottop&blast_rank=5&RID=T0HX8TYH016) |  | 71.43% |

**vgat shRNA:**

CCGGGATGATGCTGTGCGGTATATTCTCGAGAATATACCGCACAGCATCATCTTTTTG

**vmat shRNA:**

CCGGGAAACCACCACTCCCTATAATCTCGAGATTATAGGGAGTGGTGGTTTCTTTTTG


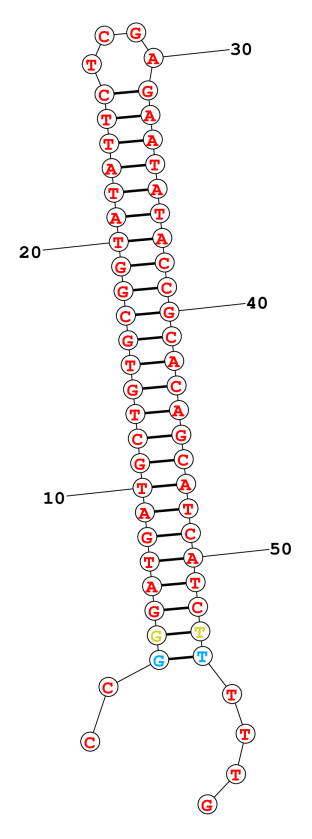

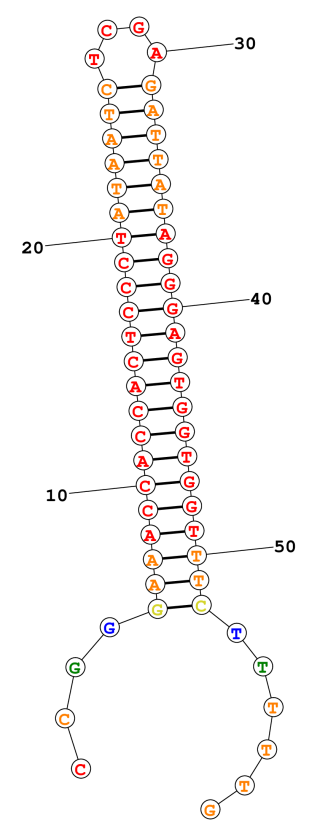


vgat vmat

Fig. S1. Sequence of *vgat* and *vmat* shRNA and predicted shRNA secondary structure


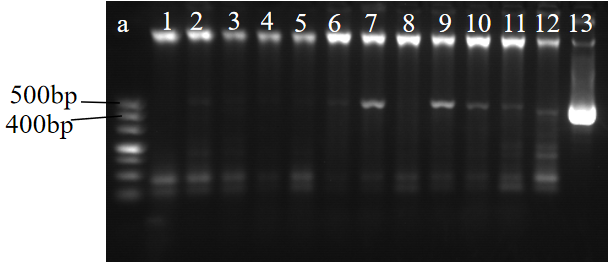

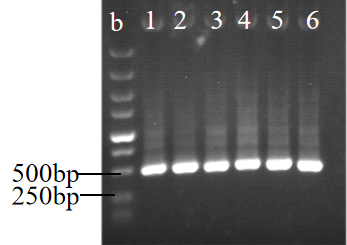


A B

Fig. S2. PCR electrophoresis results of recombinant plasmid pCAMvgat-shRNA (A) and pCAMvmat-shRNA (B) transgenic algal lines.

A, a: DL500 DNA marker; 1-13: PCR product of pCAMvgat-shRNA transformed *Chlorella*, and partially transformed algal lines. B, b: DL5000 DNA marker; 1-6: Partial PCR results of pCAMvmat-shRNA transformed *Chlorella*. Primers were designed with the 35S promoter region of the pCAMBIA1302 vector, and the resulting amplification product was 510 bp.


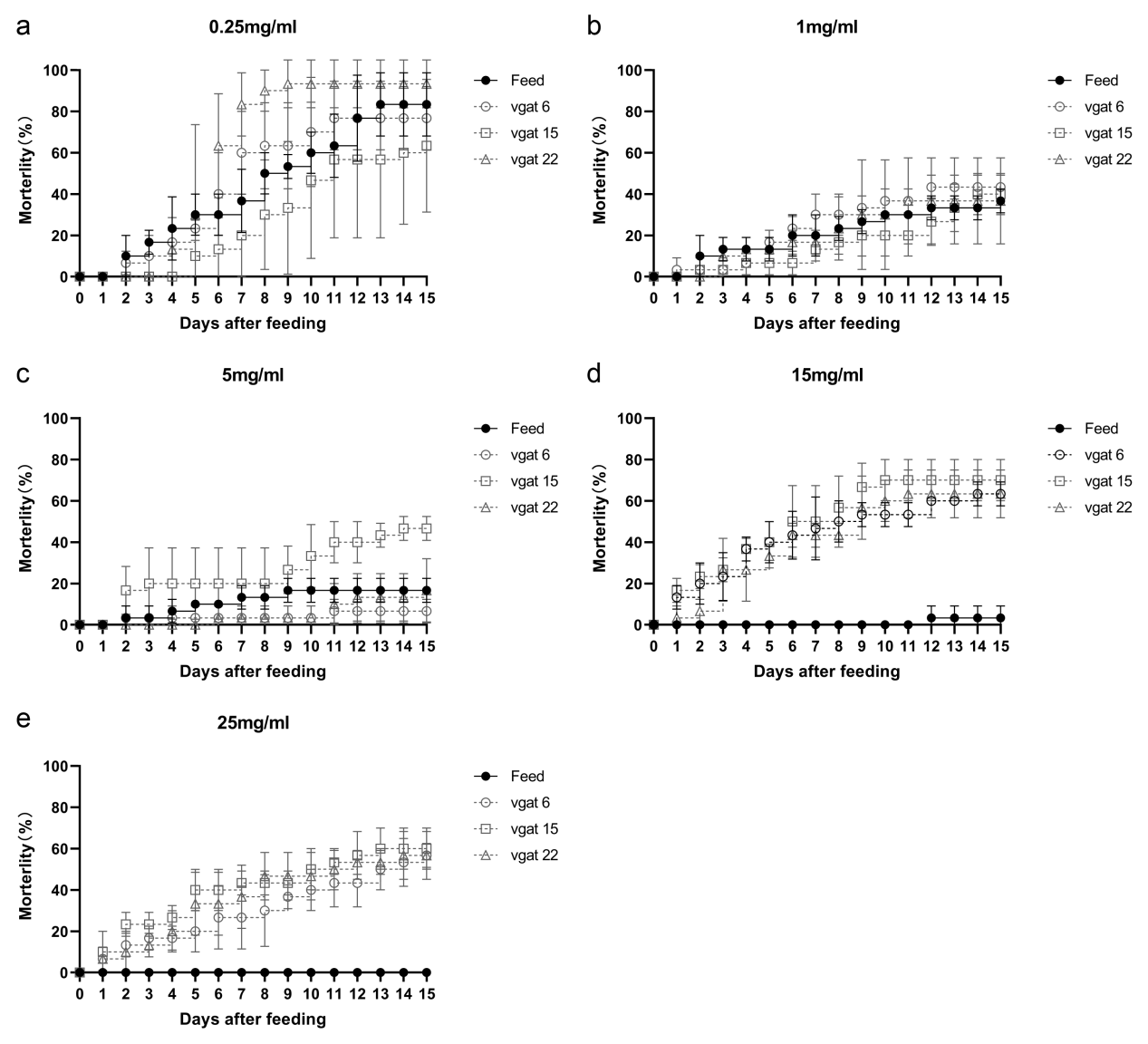


Fig. S3. Mortality of mosquito larvae fed with vgat shRNA recombinant *Chlorella*

(a) larval mortality fed 0.25 mg/ mL *vgat* shRNA recombinant *Chlorella*; (b) larval mortality fed 1 mg/mL *vgat* shRNA recombinant *Chlorella*; (c) larval mortality fed 5 mg/mL *vgat* shRNA recombinant *Chlorella*; (d) larval mortality fed 15 mg/mL *vgat* shRNA recombinant *Chlorella*; (e) larval mortality fed 25 mg/mL *vgat* shRNA recombinant *Chlorella*. Feed, larval diet fed larvae; vgat 6, vgat 15 and vgat 22, larvae fed *vgat* shRNA recombinant *Chlorella* line6, line15 and line22.


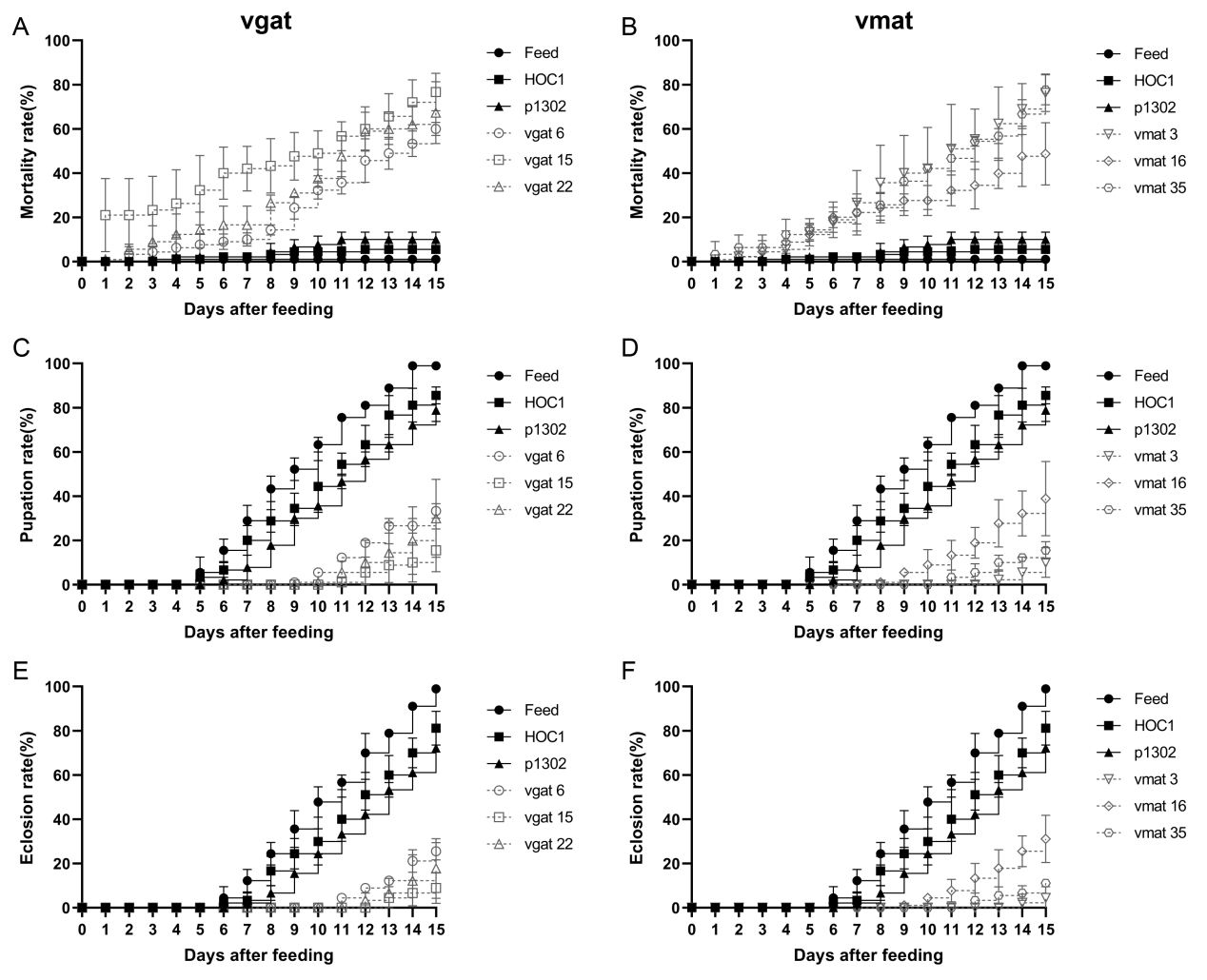


Fig. S4. Mortality, pupation and emergence rates of larvae fed *vgat* or *vmat* shRNA recombinant *Chlorella.*

(A) Larval mortality rate fed vgat shRNA recombinant *Chlorella*; (B) Larval mortality rate fed *vmat* shRNA recombinant *Chlorella*; (C) Larval pupation rate fed *vgat* shRNA recombinant *Chlorella*; (D) Larval pupation rate fed *vmat* shRNA recombinant *Chlorella*; (E) Larval eclosion rate fed *vgat* shRNA recombinant *Chlorella*; (F) Larval eclosion rate fed *vmat* shRNA recombinant *Chlorella.* Feed, larval diet fed larvae; HOC1, wild *C. vulgaris* HOC1 fed larvae; p1302, larvae fed empty plasmid pCAMBIA1302 transgenic *Chlorella*; vgat 6, vgat 15 and vgat 22, larvae fed *vgat* shRNA recombinant *Chlorella* line6, line15 and line22; vmat3, vmat16 and vmat35, larvae fed *vmat* shRNA Recombinant *Chlorella* line3, line16 and line35.


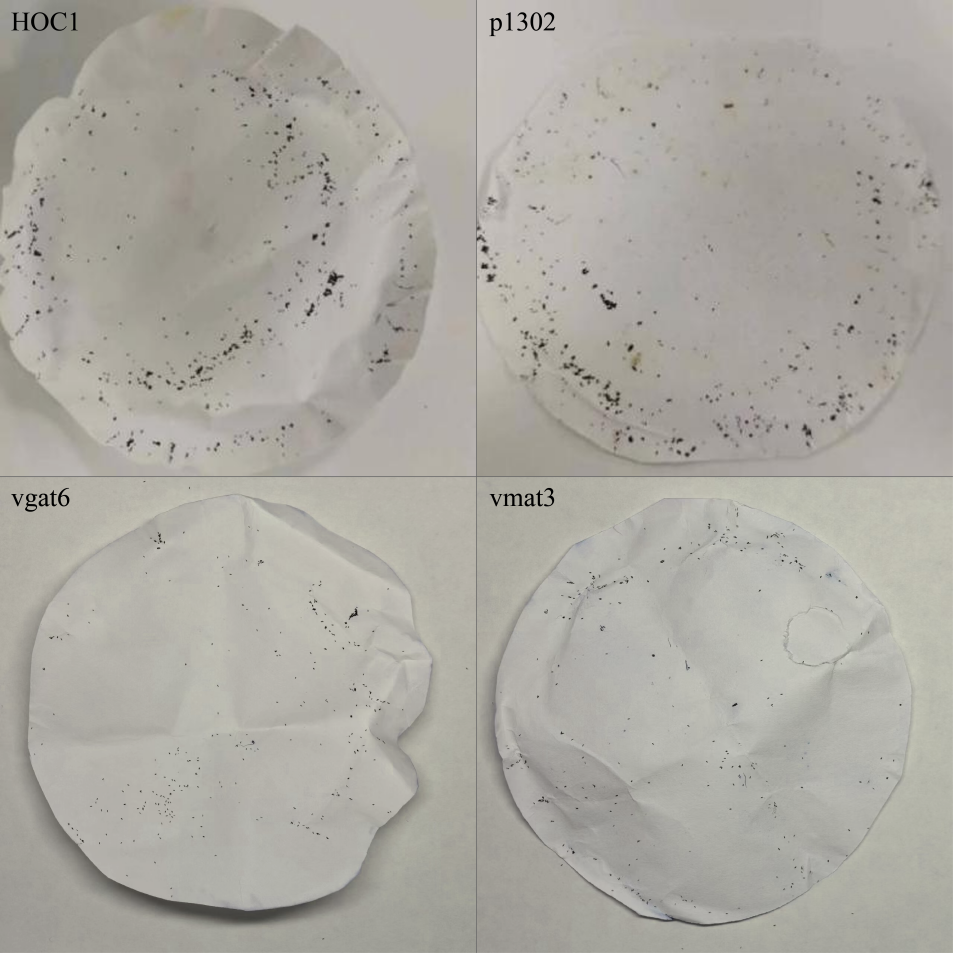


Fig. S5. Paper with eggs laid by *Ae. albopictus* in semi-field trial

HOC1, eggs laid by mosquito fed with wild *C. vulgaris* HOC1 bait; p1302, eggs laid by mosquito fed with pCAMBIA1302 transgenic *Chlorella* bait; vgat 6, eggs laid by mosquito fed with vgat shRNA recombinant *Chlorella* line 6 bait; vmat3, eggs laid by mosquito fed with vmat shRNA recombinant *Chlorella* line 3 bait.
